# Supplementary material for: Synergistic antibacterial action of AgNP-ampicillin conjugates: Evading β-lactamase degradation in ampicillin-resistant clinical isolates
Source: PLoS One. 2025 Sep 9;20(9):e0331669. doi: 10.1371/journal.pone.0331669 (PMC12419620; doi:10.1371/journal.pone.0331669)
Supplement: S1 File — S1 Figure. Standard calibration curve of pure ampicillin in distilled water at 216 nm. S1 Appendix. UV-visible Spectroscopy Data. S2 Appendix. FTIR Data. S3 Appendix. DLS and Zeta Potential Data. S4 Appendix. SEM Data. S5 Appendix. EDX Data. S6 Appendix. TGA Data. S7 Appendix. AgNP-ampicillin Synthesis Reaction. S8 Appendix. Microbiological Study Data. S9 Appendix. Molecular Docking Data. S10 Appendix. Cytotoxicity Assay Procedure. (ZIP) [file pone.0331669.s001.zip › Supporting Informations/S10_Appendix/S10_Appendix (Cytotoxicity Assay Procedure).docx]

**1. Ampicillin**

- **Plate Type**: 96-well plate (for high-throughput screening).
- **Cell Line**: Vero cells.
- **Incubation**:
  - Initial incubation of cells: Overnight (to allow cell attachment).
  - Post-treatment incubation: 24 hour.
- **Sample Preparation**:
  - Prepare serial dilutions of Ampicillin in the culture medium to determine the IC50 (e.g., 0.1 µg/mL to 100 µg/mL).
  - Add 20% of the sample directly to the wells.
- **Washing**:
  - Wash with PBS once or twice after treatment, if necessary, to remove residual Ampicillin.
- **Cytotoxicity Assay**:
  - Use the MTT assay to measure cell viability.
  - Measure absorbance at 570 nm after dissolving formazan crystals in DMSO.
- **Controls**:
  - Positive control: A known cytotoxic agent (e.g., Doxorubicin).
  - Negative control: Untreated cells.

**2. Ampicillin-AgNP Conjugates**

- **Plate Type**: 96-well plate.
- **Cell Line**: Vero cells.
- **Incubation**:
  - Initial incubation of cells: Overnight.
  - Post-treatment incubation: 24 hour.
- **Sample Preparation**:
  - Prepare serial dilutions of the Ampicillin-AgNP conjugates (e.g., 0.1 µg/mL to 100 µg/mL).
  - Add 10% or 20% of the sample directly to the wells.
- **Washing**:
  - Wash with PBS or media 1-3 times to remove unbound nanoparticles (as per the document).
- **Cytotoxicity Assay**:
  - Use the MTT assay or an alternative assay like XTT or LDH release assay for nanoparticle-based samples.
  - Measure absorbance at 570 nm (MTT) or 450 nm (XTT).
- **Controls**:
  - Positive control: A known cytotoxic agent.
  - Negative control: Untreated cells.
