# Supplementary material for: Synergistic antibacterial action of AgNP-ampicillin conjugates: Evading β-lactamase degradation in ampicillin-resistant clinical isolates
Source: PLoS One. 2025 Sep 9;20(9):e0331669. doi: 10.1371/journal.pone.0331669 (PMC12419620; doi:10.1371/journal.pone.0331669)

**UV-visible Spectroscopic Data of AgNPs**

**Reagents’ Spectra
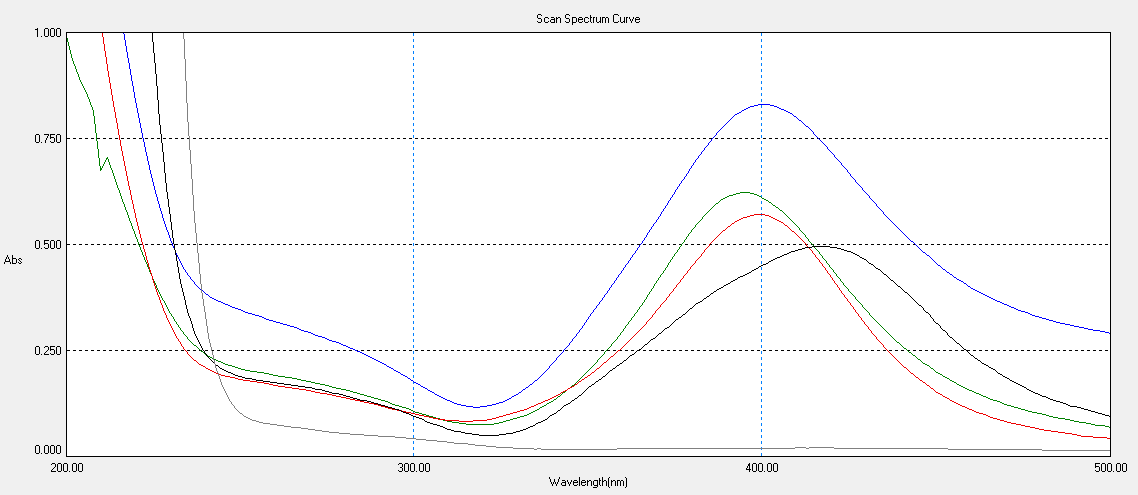
**


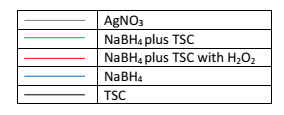


**
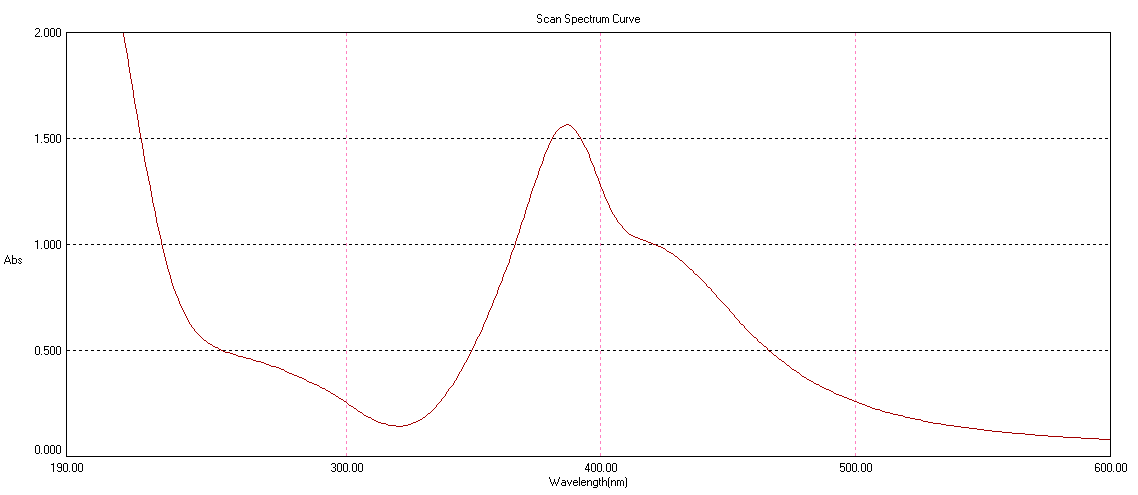
AgNPs (4X dilution)**

**AgNPs (8X dilution)**


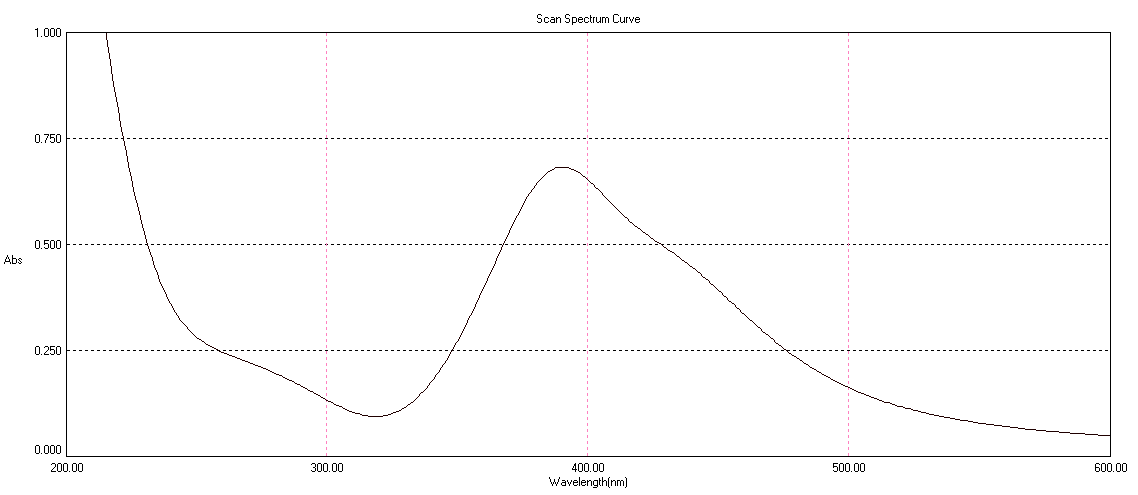


**AgNPs (16X dilution)

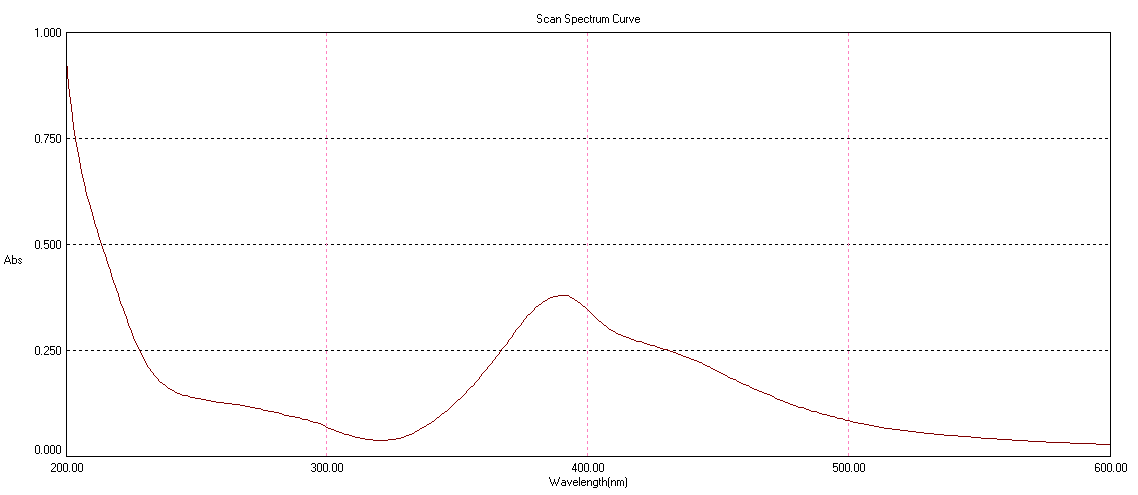
**

**AgNPs (32X dilution)**


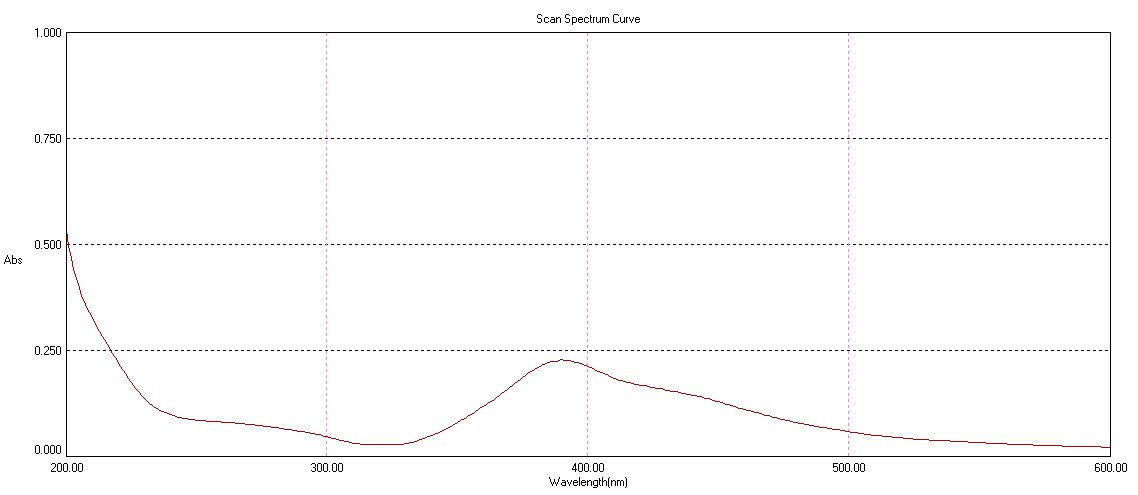

Supplement: S1 File — S1 Figure. Standard calibration curve of pure ampicillin in distilled water at 216 nm. S1 Appendix. UV-visible Spectroscopy Data. S2 Appendix. FTIR Data. S3 Appendix. DLS and Zeta Potential Data. S4 Appendix. SEM Data. S5 Appendix. EDX Data. S6 Appendix. TGA Data. S7 Appendix. AgNP-ampicillin Synthesis Reaction. S8 Appendix. Microbiological Study Data. S9 Appendix. Molecular Docking Data. S10 Appendix. Cytotoxicity Assay Procedure. (ZIP) [file pone.0331669.s001.zip › Supporting Informations/S1_Appendix (UV-visible Spectroscopy Data)/UV-visible spectroscopy data.docx]
