# Supplementary material for: Synergistic antibacterial action of AgNP-ampicillin conjugates: Evading β-lactamase degradation in ampicillin-resistant clinical isolates
Source: PLoS One. 2025 Sep 9;20(9):e0331669. doi: 10.1371/journal.pone.0331669 (PMC12419620; doi:10.1371/journal.pone.0331669)
Supplement: S1 File — S1 Figure. Standard calibration curve of pure ampicillin in distilled water at 216 nm. S1 Appendix. UV-visible Spectroscopy Data. S2 Appendix. FTIR Data. S3 Appendix. DLS and Zeta Potential Data. S4 Appendix. SEM Data. S5 Appendix. EDX Data. S6 Appendix. TGA Data. S7 Appendix. AgNP-ampicillin Synthesis Reaction. S8 Appendix. Microbiological Study Data. S9 Appendix. Molecular Docking Data. S10 Appendix. Cytotoxicity Assay Procedure. (ZIP) [file pone.0331669.s001.zip › Supporting Informations/S2_Appendix (FTIR Data)/AgNP-ampicillin.pdf]

SHIMADZU

CARS, DU

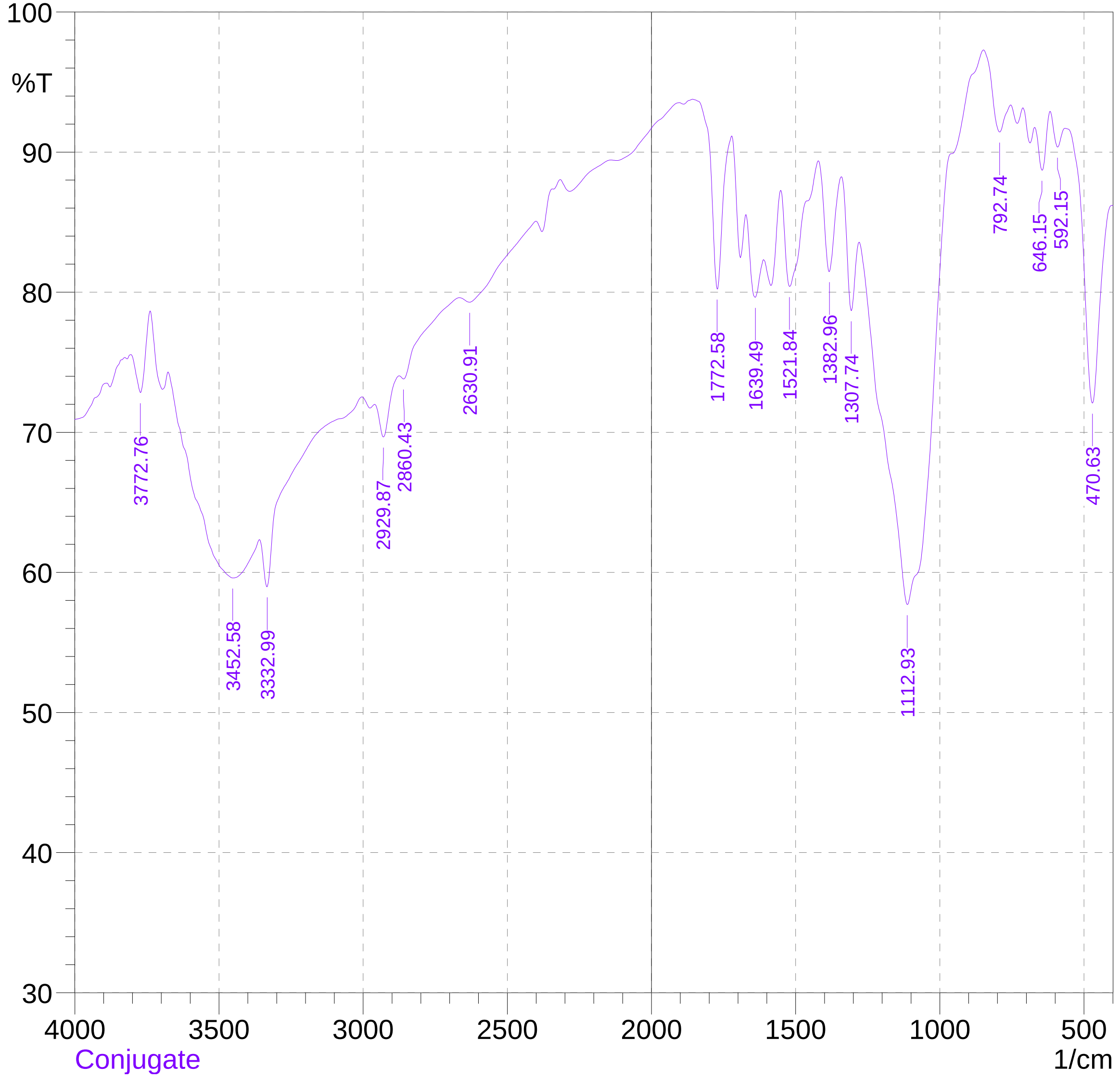

	Peak	Intensit	Corr. In	Base (H	Base (L	Area	Corr. Ar
1	470.63	72.082	16.368	565.14	405.05	13.158	4.985
2	592.15	90.358	1.949	617.22	567.07	1.983	0.239
3	646.15	88.704	3.594	671.23	617.22	2.328	0.458
4	792.74	91.436	3.532	848.68	754.17	2.68	0.707
5	1112.93	57.704	31.169	1278.81	850.61	52.152	32.948
6	1307.74	78.685	6.926	1342.46	1280.73	5.089	1.006
7	1382.96	81.474	7.351	1421.54	1342.46	5.464	1.388
8	1521.84	80.412	6.606	1552.7	1460.11	7.296	1.647
9	1639.49	79.639	4.137	1672.28	1612.49	5.297	0.744
10	1772.58	80.228	11.898	1857.45	1722.43	6.864	2.263
11	2630.91	79.287	1.038	2665.62	2401.38	23.092	0.754
12	2860.43	73.811	0.587	2873.94	2667.55	23.107	0.076
13	2929.87	69.663	3.041	2958.8	2875.86	11.949	0.63
14	3332.99	58.98	4.06	3358.07	3005.1	59.015	0.726
15	3452.58	59.602	6.24	3676.32	3360	62.138	9.203
16	3772.76	72.834	4.23	3805.55	3738.05	8.446	0.818

Comment;
Conjugate

Date/Time; 1/19/2020 12:44:28 PM
No. of Scans;
Resolution;
Apodization;
