# Supplementary material for: Synergistic antibacterial action of AgNP-ampicillin conjugates: Evading β-lactamase degradation in ampicillin-resistant clinical isolates
Source: PLoS One. 2025 Sep 9;20(9):e0331669. doi: 10.1371/journal.pone.0331669 (PMC12419620; doi:10.1371/journal.pone.0331669)
Supplement: S1 File — S1 Figure. Standard calibration curve of pure ampicillin in distilled water at 216 nm. S1 Appendix. UV-visible Spectroscopy Data. S2 Appendix. FTIR Data. S3 Appendix. DLS and Zeta Potential Data. S4 Appendix. SEM Data. S5 Appendix. EDX Data. S6 Appendix. TGA Data. S7 Appendix. AgNP-ampicillin Synthesis Reaction. S8 Appendix. Microbiological Study Data. S9 Appendix. Molecular Docking Data. S10 Appendix. Cytotoxicity Assay Procedure. (ZIP) [file pone.0331669.s001.zip › Supporting Informations/S2_Appendix (FTIR Data)/AgNPs.pdf]

SHIMADZU

CARS, DU

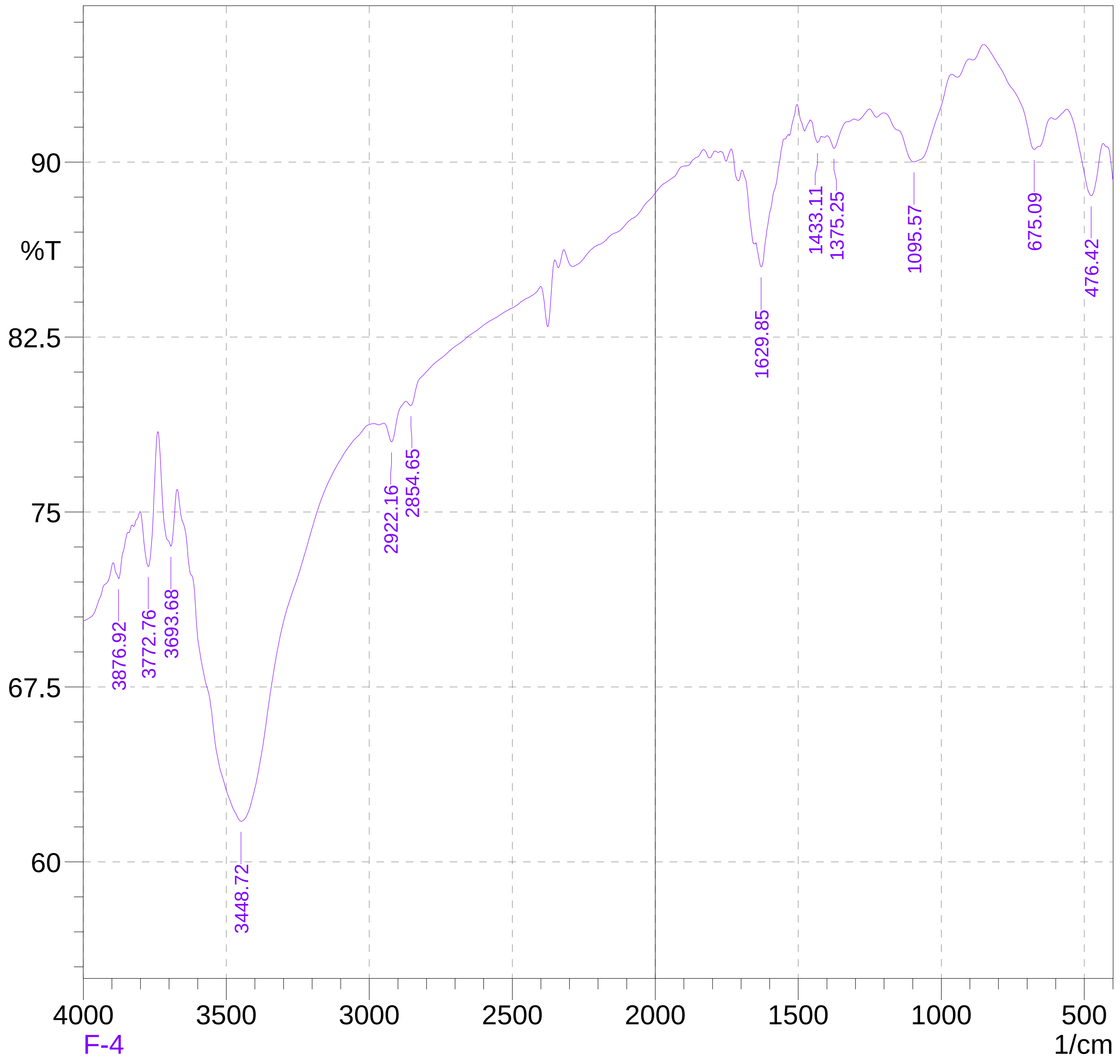

	Peak	Intensit	Corr. In	Base (H	Base (L	Area	Corr. Ar
1	476.42	88.56	2.731	561.29	433.98	5.686	0.795
2	675.09	90.541	2.136	852.54	617.22	7.742	0.832
3	1095.57	90.01	2.842	1201.65	964.41	9.32	1.771
4	1375.25	90.59	0.749	1400.32	1327.03	2.953	0.103
5	1433.11	90.841	0.513	1458.18	1419.61	1.542	0.046
6	1629.85	85.512	1.831	1647.21	1550.77	5.415	0.412
7	2854.65	79.56	0.366	2872.01	2401.38	39.321	-0.822
8	2922.16	78.005	1.13	2949.16	2872.01	7.959	0.177
9	3448.72	61.742	15.145	3672.47	2983.88	104.329	27.59
10	3693.68	73.53	3.218	3739.97	3672.47	8.415	0.827
11	3772.76	72.659	3.897	3799.77	3739.97	7.645	0.777
12	3876.92	72.143	1.138	3894.28	3846.06	6.612	0.16

Comment;

F-4

Date/Time; 12/12/2018 12:01:07 PM

No. of Scans;

Resolution;

Apodization;
