# Supplementary material for: Synergistic antibacterial action of AgNP-ampicillin conjugates: Evading β-lactamase degradation in ampicillin-resistant clinical isolates
Source: PLoS One. 2025 Sep 9;20(9):e0331669. doi: 10.1371/journal.pone.0331669 (PMC12419620; doi:10.1371/journal.pone.0331669)
Supplement: S1 File — S1 Figure. Standard calibration curve of pure ampicillin in distilled water at 216 nm. S1 Appendix. UV-visible Spectroscopy Data. S2 Appendix. FTIR Data. S3 Appendix. DLS and Zeta Potential Data. S4 Appendix. SEM Data. S5 Appendix. EDX Data. S6 Appendix. TGA Data. S7 Appendix. AgNP-ampicillin Synthesis Reaction. S8 Appendix. Microbiological Study Data. S9 Appendix. Molecular Docking Data. S10 Appendix. Cytotoxicity Assay Procedure. (ZIP) [file pone.0331669.s001.zip › Supporting Informations/S2_Appendix (FTIR Data)/Ampicillin.pdf]

SHIMADZU

CARS, DU

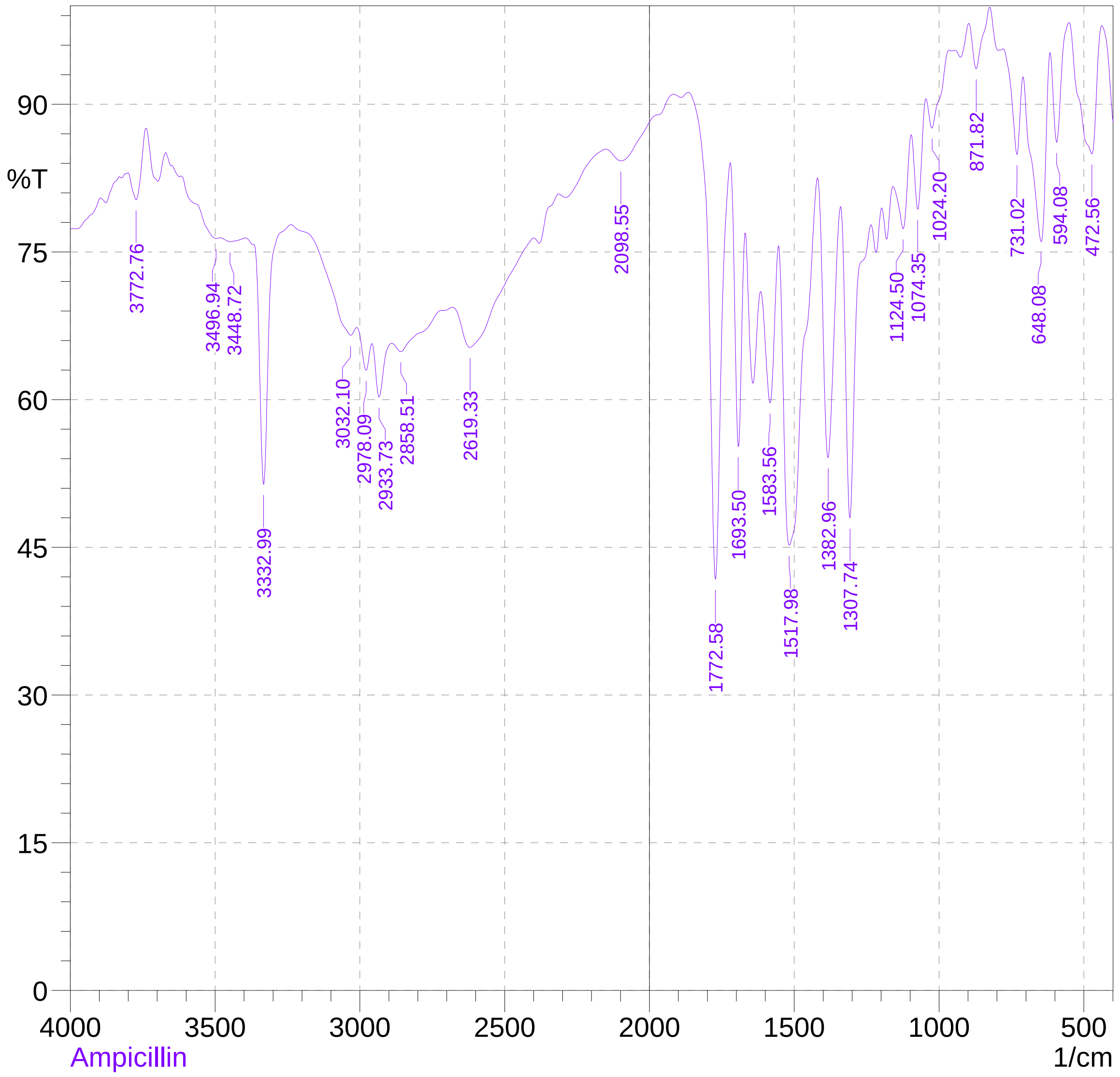

	Peak	Intensit	Corr. In	Base (H	Base (L	Area	Corr. Ar
1	472.56	84.954	13.134	551.64	437.84	4.889	3.965
2	594.08	86.127	10.197	617.22	551.64	2.226	1.288
3	648.08	76.053	18.376	709.8	617.22	7.064	4.582
4	731.02	84.897	8.769	779.24	709.8	2.972	1.172
5	871.82	93.615	5.177	896.9	825.53	1.117	0.817
6	1024.2	87.579	4.29	1045.42	964.41	3.384	0.834
7	1074.35	79.345	9.102	1095.57	1045.42	3.782	1.173
8	1124.5	77.356	7.144	1159.22	1095.57	5.91	1.163
9	1307.74	47.982	30.973	1338.6	1236.37	17.993	7.293
10	1382.96	54.113	27.054	1419.61	1340.53	13.749	6.517
11	1517.98	45.237	32.057	1552.7	1421.54	29.063	15.568
12	1583.56	59.667	13.678	1614.42	1554.63	10.788	2.724
13	1693.5	55.238	24.979	1720.5	1670.35	8.47	3.722
14	1772.58	41.743	44.781	1865.17	1722.43	20.194	11.91
15	2098.55	84.249	2.392	2148.7	1917.24	14.051	1.439
16	2619.33	65.303	5.58	2679.13	2399.45	43.147	4.655
17	2858.51	64.874	1.444	2889.37	2717.7	30.224	0.805
18	2933.73	60.269	5.387	2956.87	2891.3	13.007	1.03
19	2978.09	62.97	3.326	3010.88	2958.8	9.78	0.545
20	3032.1	66.534	1.676	3236.55	3012.81	31.085	0.61
21	3332.99	51.387	24.893	3365.78	3238.48	19.717	5.106
22	3448.72	76.038	0.391	3481.51	3396.64	10.014	0.105
23	3496.94	76.366	0.692	3618.46	3483.44	13.942	0.468
24	3772.76	80.29	4.811	3801.7	3738.05	5.235	0.833

Comment;

Ampicillin

Date/Time; 1/19/2020 12:35:48 PM

No. of Scans;

Resolution;

Apodization;
