# Supplementary material for: Synergistic antibacterial action of AgNP-ampicillin conjugates: Evading β-lactamase degradation in ampicillin-resistant clinical isolates
Source: PLoS One. 2025 Sep 9;20(9):e0331669. doi: 10.1371/journal.pone.0331669 (PMC12419620; doi:10.1371/journal.pone.0331669)

Size Distribution Report by Intensity

v2.2

Malvern

Sample Details

**Sample Name:**	C4 1
**SOP Name:**	mansettings.nano
**General Notes:**	

**File Name:**	Nadia.dts	**Dispersant Name:**	Ethylene Glycol
**Record Number:**	21	**Dispersant RI:**	1.429
**Material RI:**	1.59	**Viscosity (cP):**	16.1118
**Material Absorbtion:**	0.010	**Measurement Date and Time:**	Wednesday, December 12, 20...

System

**Temperature (°C):**	25.0	**Duration Used (s):**	80
**Count Rate (kcps):**	130.4	**Measurement Position (mm):**	4.65
**Cell Description:**	Disposable sizing cuvette	**Attenuator:**	7

Results

			**Size (d.nm):**	**% Intensity:**	**St Dev (d.n...**
**Z-Average (d.nm):**	578.6	**Peak 1:**	605.3	100.0	112.4
**PdI:**	0.092	**Peak 2:**	0.000	0.0	0.000
**Intercept:**	0.917	**Peak 3:**	0.000	0.0	0.000

Result quality : **Refer to quality report**

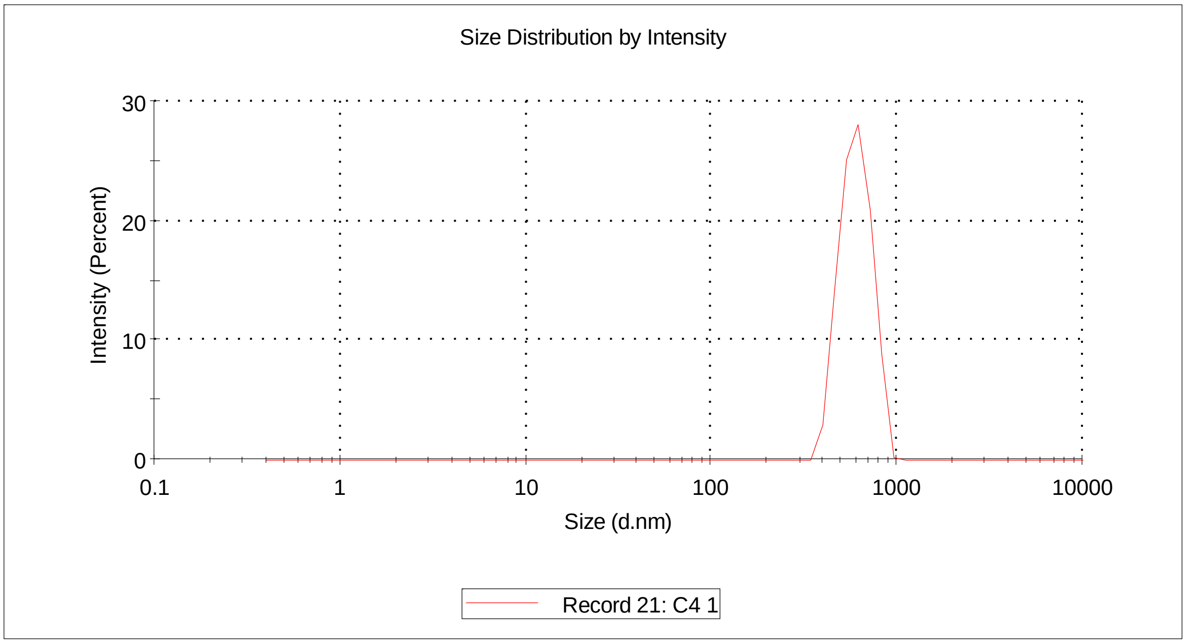

Supplement: S1 File — S1 Figure. Standard calibration curve of pure ampicillin in distilled water at 216 nm. S1 Appendix. UV-visible Spectroscopy Data. S2 Appendix. FTIR Data. S3 Appendix. DLS and Zeta Potential Data. S4 Appendix. SEM Data. S5 Appendix. EDX Data. S6 Appendix. TGA Data. S7 Appendix. AgNP-ampicillin Synthesis Reaction. S8 Appendix. Microbiological Study Data. S9 Appendix. Molecular Docking Data. S10 Appendix. Cytotoxicity Assay Procedure. (ZIP) [file pone.0331669.s001.zip › Supporting Informations/S3_Appendix (DLS and Zeta Potential Data)/Particle Size (AgNP@SiO2-NH2).pdf]
