# Supplementary material for: Synergistic antibacterial action of AgNP-ampicillin conjugates: Evading β-lactamase degradation in ampicillin-resistant clinical isolates
Source: PLoS One. 2025 Sep 9;20(9):e0331669. doi: 10.1371/journal.pone.0331669 (PMC12419620; doi:10.1371/journal.pone.0331669)

Size Distribution Report by Intensity

v2.2

Malvern

Sample Details

Sample Name: F4 1

SOP Name: mansettings.nano

General Notes:

**File Name:**	Nadia.dts	**Dispersant Name:**	Water
**Record Number:**	4	**Dispersant RI:**	1.330
**Material RI:**	1.59	**Viscosity (cP):**	0.8872
**Material Absorbtion:**	0.010	**Measurement Date and Time:**	Tuesday, December 04, 2018 ...

System

**Temperature (°C):**	25.0	**Duration Used (s):**	70
**Count Rate (kcps):**	206.0	**Measurement Position (mm):**	4.65
**Cell Description:**	Disposable sizing cuvette	**Attenuator:**	8

Results

			Size (d.nm):	% Intensity:	St Dev (d.n...
**Z-Average (d.nm):**	67.34	**Peak 1:**	30.91	56.0	17.53
**PdI:**	0.316	**Peak 2:**	202.1	34.0	89.31
**Intercept:**	0.874	**Peak 3:**	4.757	6.6	1.297

Result quality : Refer to quality report

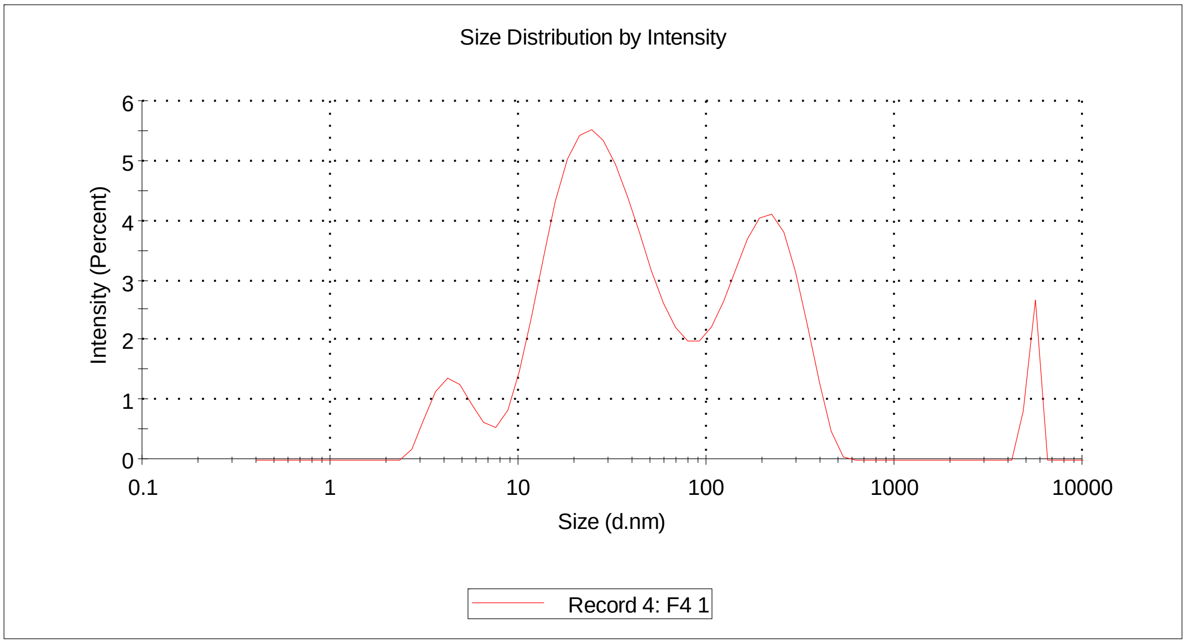

Supplement: S1 File — S1 Figure. Standard calibration curve of pure ampicillin in distilled water at 216 nm. S1 Appendix. UV-visible Spectroscopy Data. S2 Appendix. FTIR Data. S3 Appendix. DLS and Zeta Potential Data. S4 Appendix. SEM Data. S5 Appendix. EDX Data. S6 Appendix. TGA Data. S7 Appendix. AgNP-ampicillin Synthesis Reaction. S8 Appendix. Microbiological Study Data. S9 Appendix. Molecular Docking Data. S10 Appendix. Cytotoxicity Assay Procedure. (ZIP) [file pone.0331669.s001.zip › Supporting Informations/S3_Appendix (DLS and Zeta Potential Data)/Particle Size (AgNPs).pdf]
