# Supplementary material for: Synergistic antibacterial action of AgNP-ampicillin conjugates: Evading β-lactamase degradation in ampicillin-resistant clinical isolates
Source: PLoS One. 2025 Sep 9;20(9):e0331669. doi: 10.1371/journal.pone.0331669 (PMC12419620; doi:10.1371/journal.pone.0331669)
Supplement: S1 File — S1 Figure. Standard calibration curve of pure ampicillin in distilled water at 216 nm. S1 Appendix. UV-visible Spectroscopy Data. S2 Appendix. FTIR Data. S3 Appendix. DLS and Zeta Potential Data. S4 Appendix. SEM Data. S5 Appendix. EDX Data. S6 Appendix. TGA Data. S7 Appendix. AgNP-ampicillin Synthesis Reaction. S8 Appendix. Microbiological Study Data. S9 Appendix. Molecular Docking Data. S10 Appendix. Cytotoxicity Assay Procedure. (ZIP) [file pone.0331669.s001.zip › Supporting Informations/S3_Appendix (DLS and Zeta Potential Data)/Zeta Potential (AgNP-ampicillin).pdf]

Zeta Potential - Quality

Malvern Panalytical

Sample Details

**Sample Name:**	Sample 4
**Project Name:**	Project 3-2024
**Date and Time:**	Tuesday, April 30 2024 11:35:16 AM

**Type:**	Zeta	**Result Source:**	Instrument
**Cell Name:**	DTS1070	**Temperature (°C):**	25.01
**Material Name:**	Polystyrene latex	**Dispersant Name:**	Water
**Material RI:**	1.59	**Dispersant RI:**	1.33
**Material Absorption:**	0.01	**Dispersant Viscosity (cP):**	0.887
		**Dispersant Dielectric Constant:**	78.5

Zeta Potential Distribution

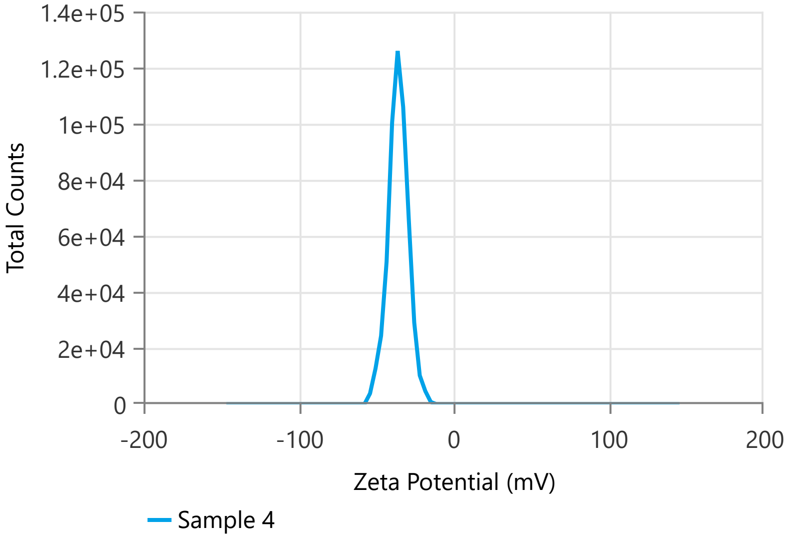

Phase Plot

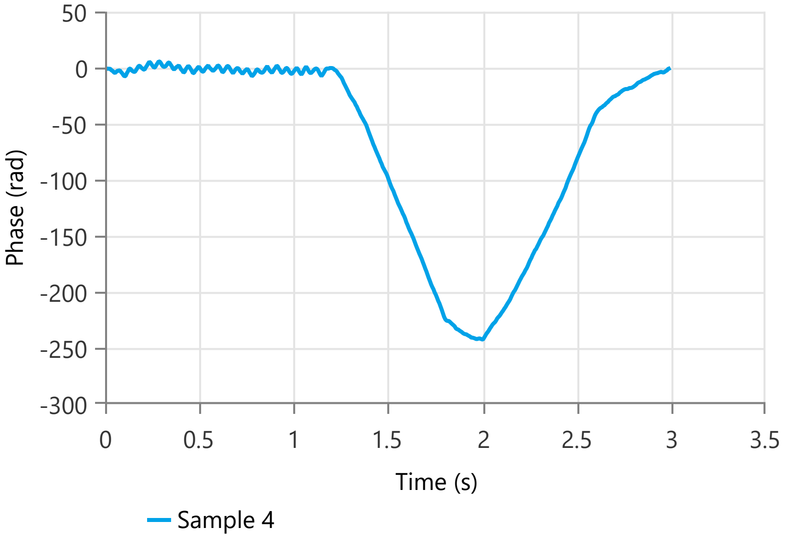

Statistics Table

Name	Mean	Standard Deviation	RSD	Minimum	Maximum
Zeta Potential (mV)	-35.7	-	-	-35.7	-35.7
Zeta Peak One Mean	-36	-	-	-36	-36
Conductivity	3.725	-	-	3.725	3.725
Wall Zeta Potential (mV)	-26.36	-	-	-26.36	-26.36
Zeta Deviation (mV)	6.46	-	-	6.46	6.46
Derived Mean Count Rate (kcps)	1.89E+04	-	-	1.89E+04	1.89E+04
Reference Beam Count Rate (kcps)	1823	-	-	1823	1823
Quality Factor	1.763	-	-	1.763	1.763

Parameter List

**Instrument Serial Number**	: MAL1232437
**Software Version**	: 1.3.0.140
