# Supplementary material for: Synergistic antibacterial action of AgNP-ampicillin conjugates: Evading β-lactamase degradation in ampicillin-resistant clinical isolates
Source: PLoS One. 2025 Sep 9;20(9):e0331669. doi: 10.1371/journal.pone.0331669 (PMC12419620; doi:10.1371/journal.pone.0331669)

Zeta Potential Report

v2.3

Malvern Instruments Ltd - © Copyright 2008

Sample Details

Sample Name: F4 1

SOP Name: mansettings.nano

General Notes:

File Name: Nadia.dts

Record Number: 12

Date and Time: Tuesday, December 04, 2018 6:0...

Dispersant Name: Water

Dispersant RI: 1.330

Viscosity (cP): 0.8872

Dispersant Dielectric Constant: 78.5

System

Temperature (°C): 25.0

Count Rate (kcps): 35.0

Cell Description: Clear disposable zeta cell

Zeta Runs: 12

Measurement Position (mm): 2.00

Attenuator: 7

Results

			Mean (mV)	Area (%)	St Dev (mV)
**Zeta Potential (mV):**	-36.7	**Peak 1:**	-21.3	47.3	10.8
**Zeta Deviation (mV):**	21.5	**Peak 2:**	-58.3	36.7	6.62
**Conductivity (mS/cm):**	0.719	**Peak 3:**	-41.9	14.6	3.59

Result quality : See result quality report

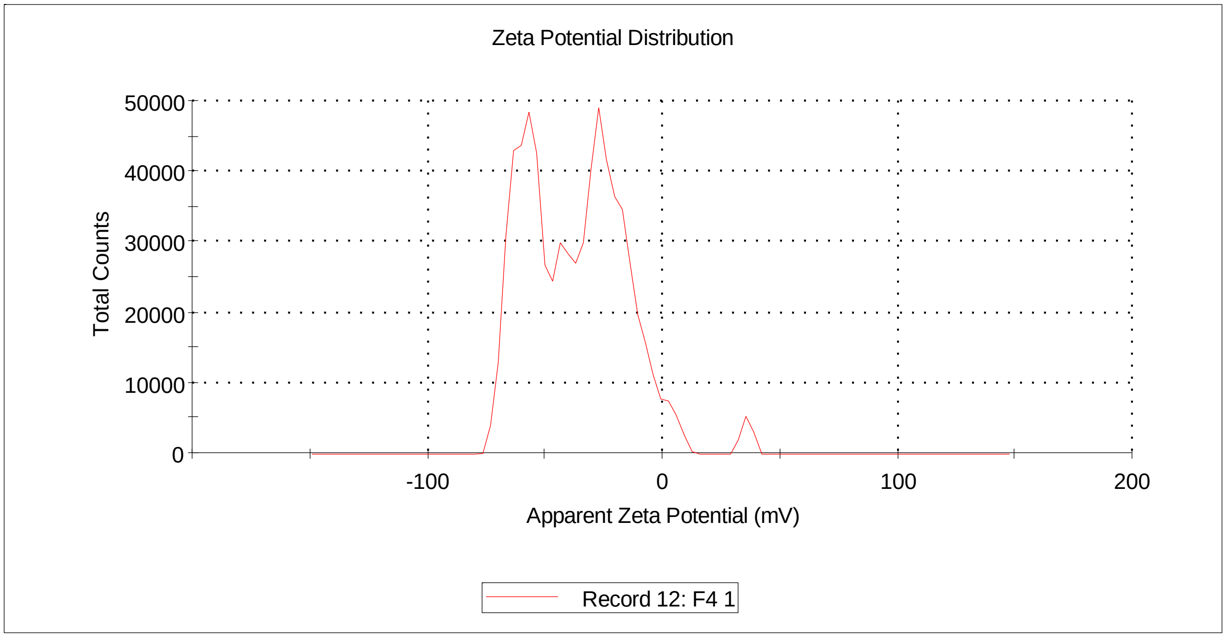

Supplement: S1 File — S1 Figure. Standard calibration curve of pure ampicillin in distilled water at 216 nm. S1 Appendix. UV-visible Spectroscopy Data. S2 Appendix. FTIR Data. S3 Appendix. DLS and Zeta Potential Data. S4 Appendix. SEM Data. S5 Appendix. EDX Data. S6 Appendix. TGA Data. S7 Appendix. AgNP-ampicillin Synthesis Reaction. S8 Appendix. Microbiological Study Data. S9 Appendix. Molecular Docking Data. S10 Appendix. Cytotoxicity Assay Procedure. (ZIP) [file pone.0331669.s001.zip › Supporting Informations/S3_Appendix (DLS and Zeta Potential Data)/Zeta Potential (AgNPs).pdf]
