# Supplementary figures and images for: Synergistic antibacterial action of AgNP-ampicillin conjugates: Evading β-lactamase degradation in ampicillin-resistant clinical isolates
Source: PLoS One. 2025 Sep 9;20(9):e0331669. doi: 10.1371/journal.pone.0331669 (PMC12419620; doi:10.1371/journal.pone.0331669)

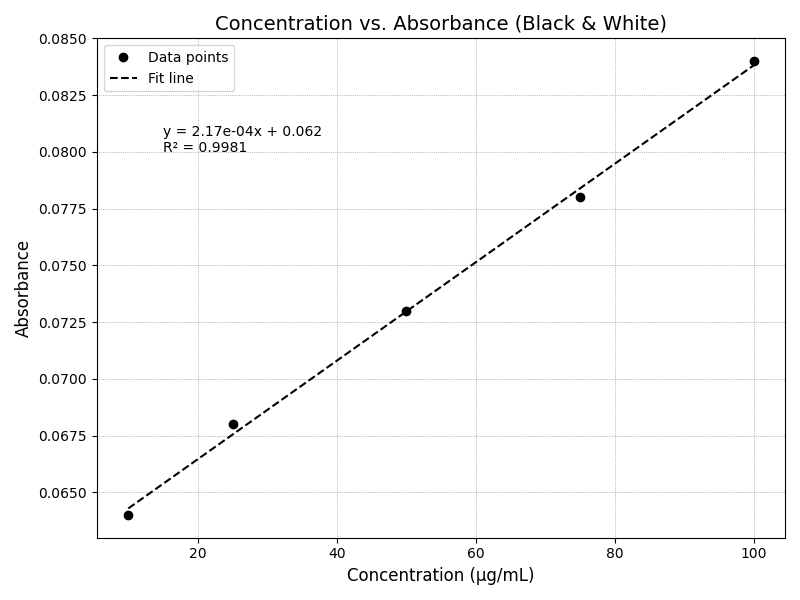

Supplement: S1 File — S1 Figure. Standard calibration curve of pure ampicillin in distilled water at 216 nm. S1 Appendix. UV-visible Spectroscopy Data. S2 Appendix. FTIR Data. S3 Appendix. DLS and Zeta Potential Data. S4 Appendix. SEM Data. S5 Appendix. EDX Data. S6 Appendix. TGA Data. S7 Appendix. AgNP-ampicillin Synthesis Reaction. S8 Appendix. Microbiological Study Data. S9 Appendix. Molecular Docking Data. S10 Appendix. Cytotoxicity Assay Procedure. (ZIP) [file pone.0331669.s001.zip › Supporting Informations/S1_Figure/S1_Figure.tif]

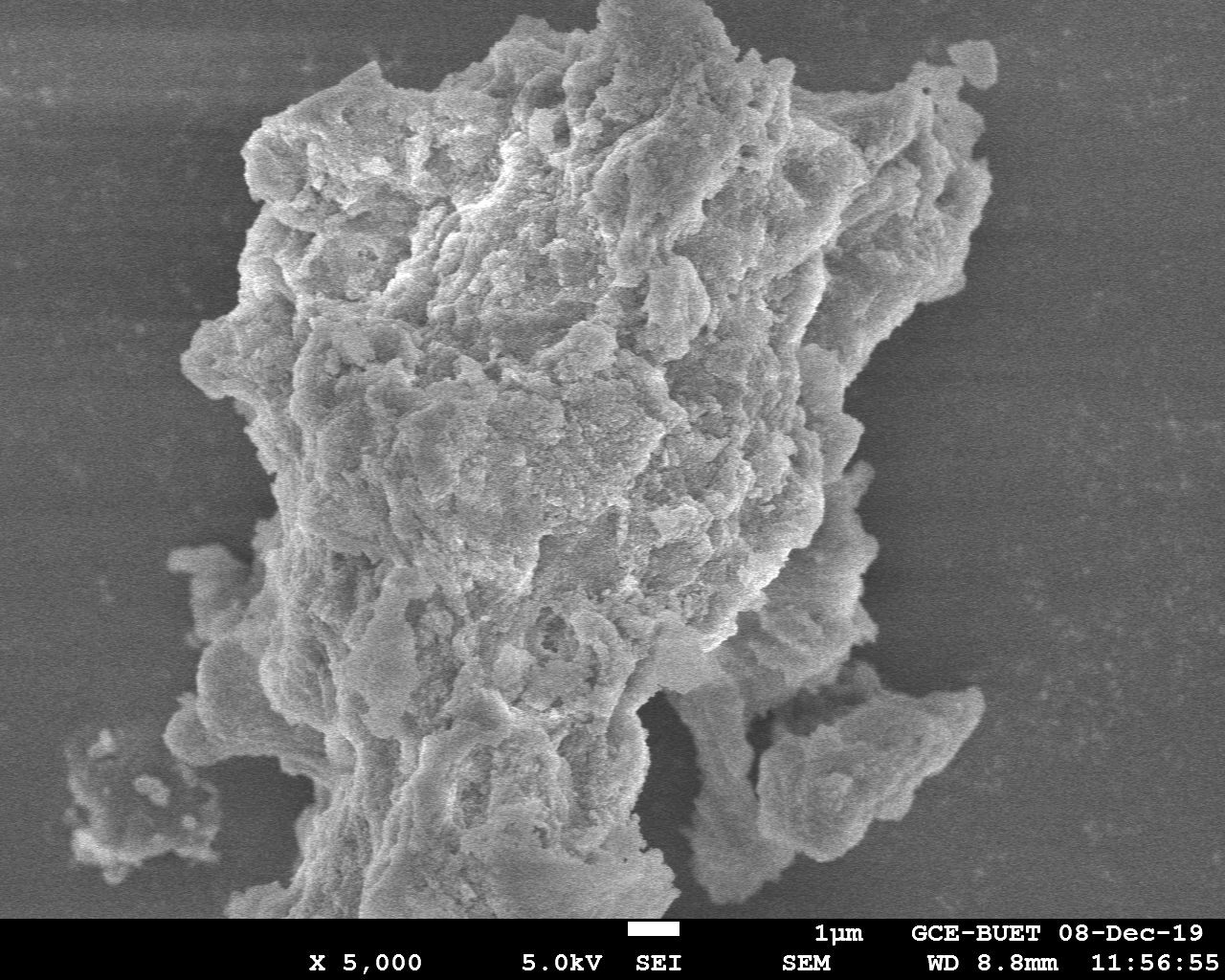

Supplement: S1 File — S1 Figure. Standard calibration curve of pure ampicillin in distilled water at 216 nm. S1 Appendix. UV-visible Spectroscopy Data. S2 Appendix. FTIR Data. S3 Appendix. DLS and Zeta Potential Data. S4 Appendix. SEM Data. S5 Appendix. EDX Data. S6 Appendix. TGA Data. S7 Appendix. AgNP-ampicillin Synthesis Reaction. S8 Appendix. Microbiological Study Data. S9 Appendix. Molecular Docking Data. S10 Appendix. Cytotoxicity Assay Procedure. (ZIP) [file pone.0331669.s001.zip › Supporting Informations/S4_Appendix (SEM Data)/AgNP-ampicillin.jpg]

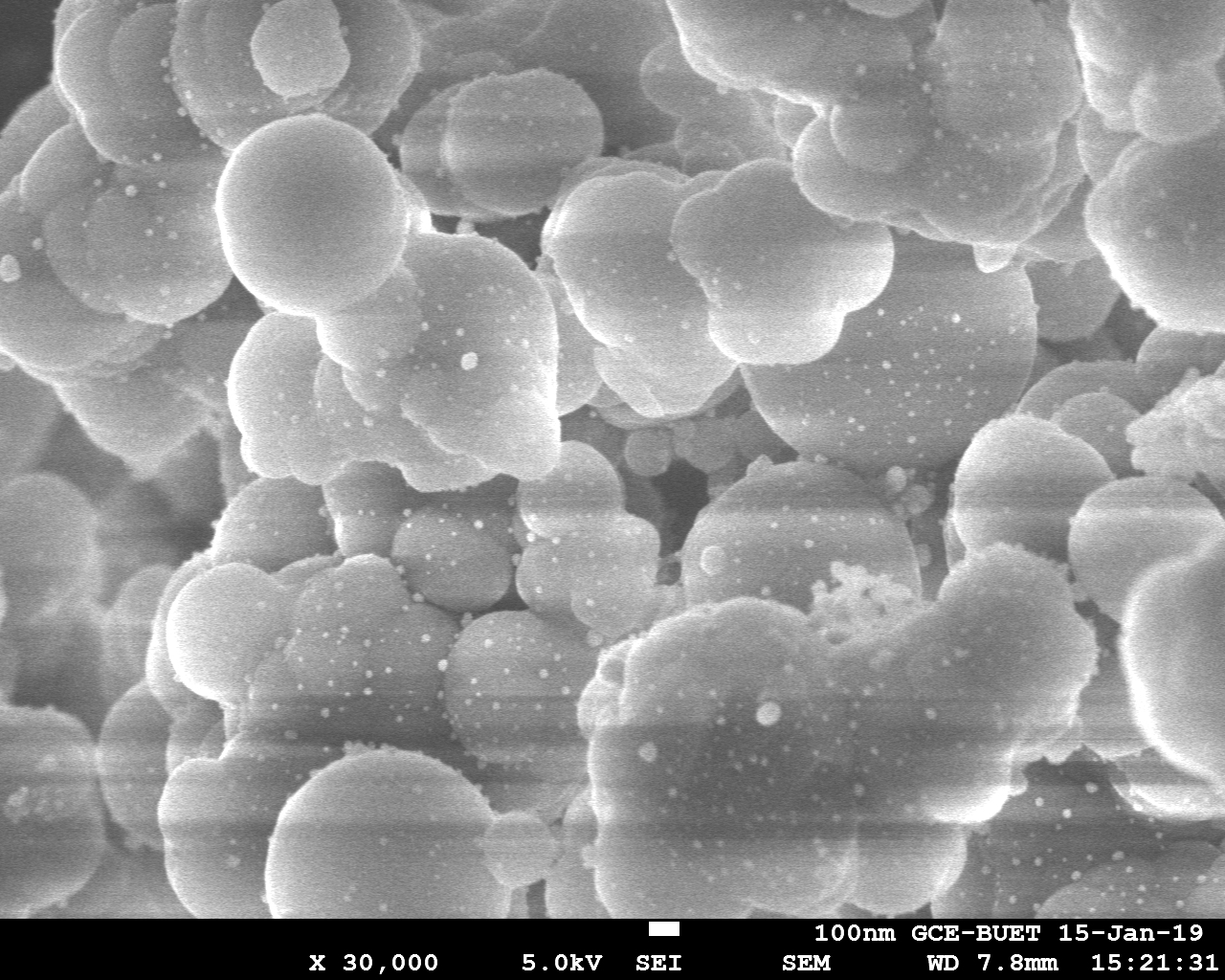

Supplement: S1 File — S1 Figure. Standard calibration curve of pure ampicillin in distilled water at 216 nm. S1 Appendix. UV-visible Spectroscopy Data. S2 Appendix. FTIR Data. S3 Appendix. DLS and Zeta Potential Data. S4 Appendix. SEM Data. S5 Appendix. EDX Data. S6 Appendix. TGA Data. S7 Appendix. AgNP-ampicillin Synthesis Reaction. S8 Appendix. Microbiological Study Data. S9 Appendix. Molecular Docking Data. S10 Appendix. Cytotoxicity Assay Procedure. (ZIP) [file pone.0331669.s001.zip › Supporting Informations/S4_Appendix (SEM Data)/AgNP@SiO2-NH2.jpg]

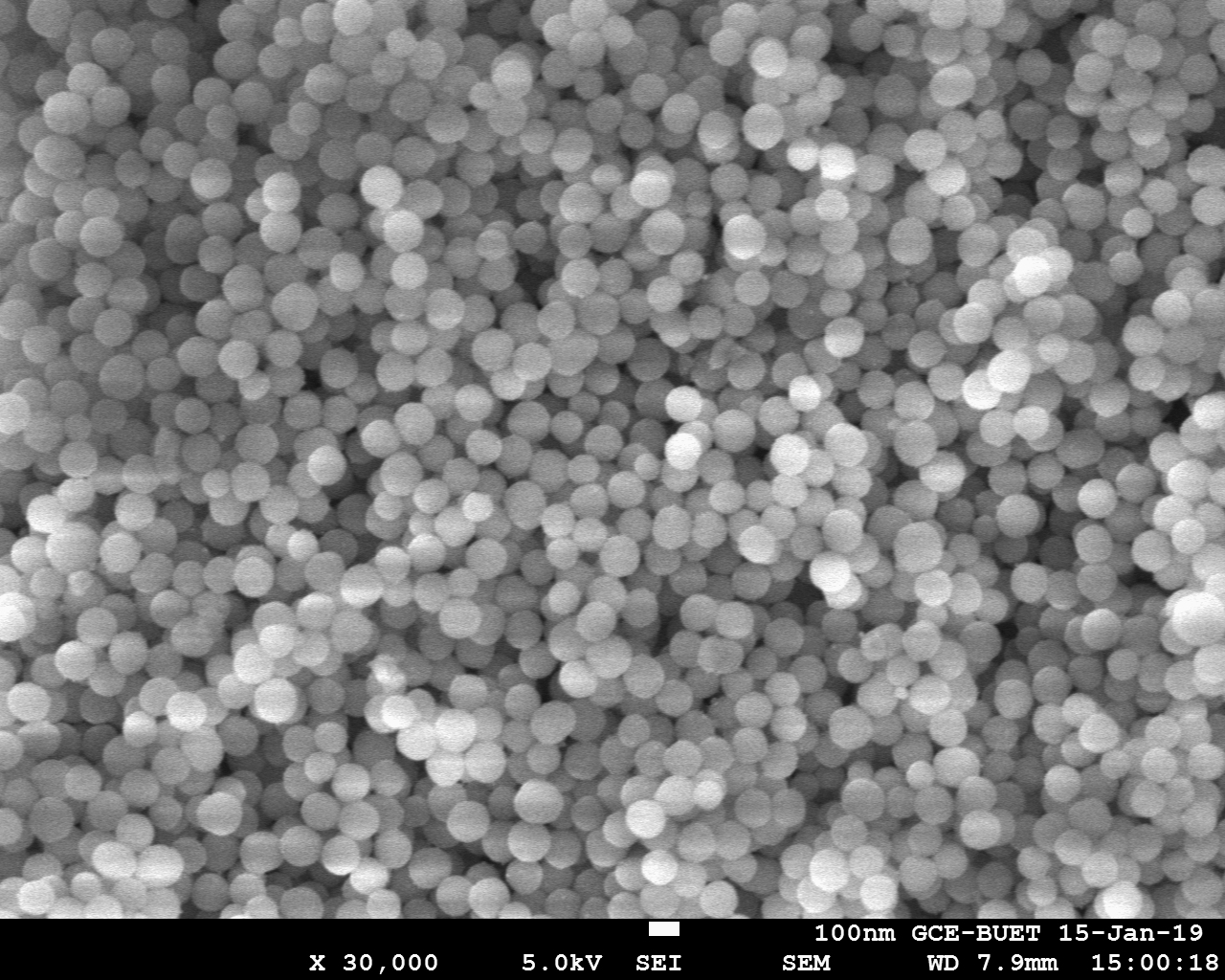

Supplement: S1 File — S1 Figure. Standard calibration curve of pure ampicillin in distilled water at 216 nm. S1 Appendix. UV-visible Spectroscopy Data. S2 Appendix. FTIR Data. S3 Appendix. DLS and Zeta Potential Data. S4 Appendix. SEM Data. S5 Appendix. EDX Data. S6 Appendix. TGA Data. S7 Appendix. AgNP-ampicillin Synthesis Reaction. S8 Appendix. Microbiological Study Data. S9 Appendix. Molecular Docking Data. S10 Appendix. Cytotoxicity Assay Procedure. (ZIP) [file pone.0331669.s001.zip › Supporting Informations/S4_Appendix (SEM Data)/AgNP@SiO2.jpg]

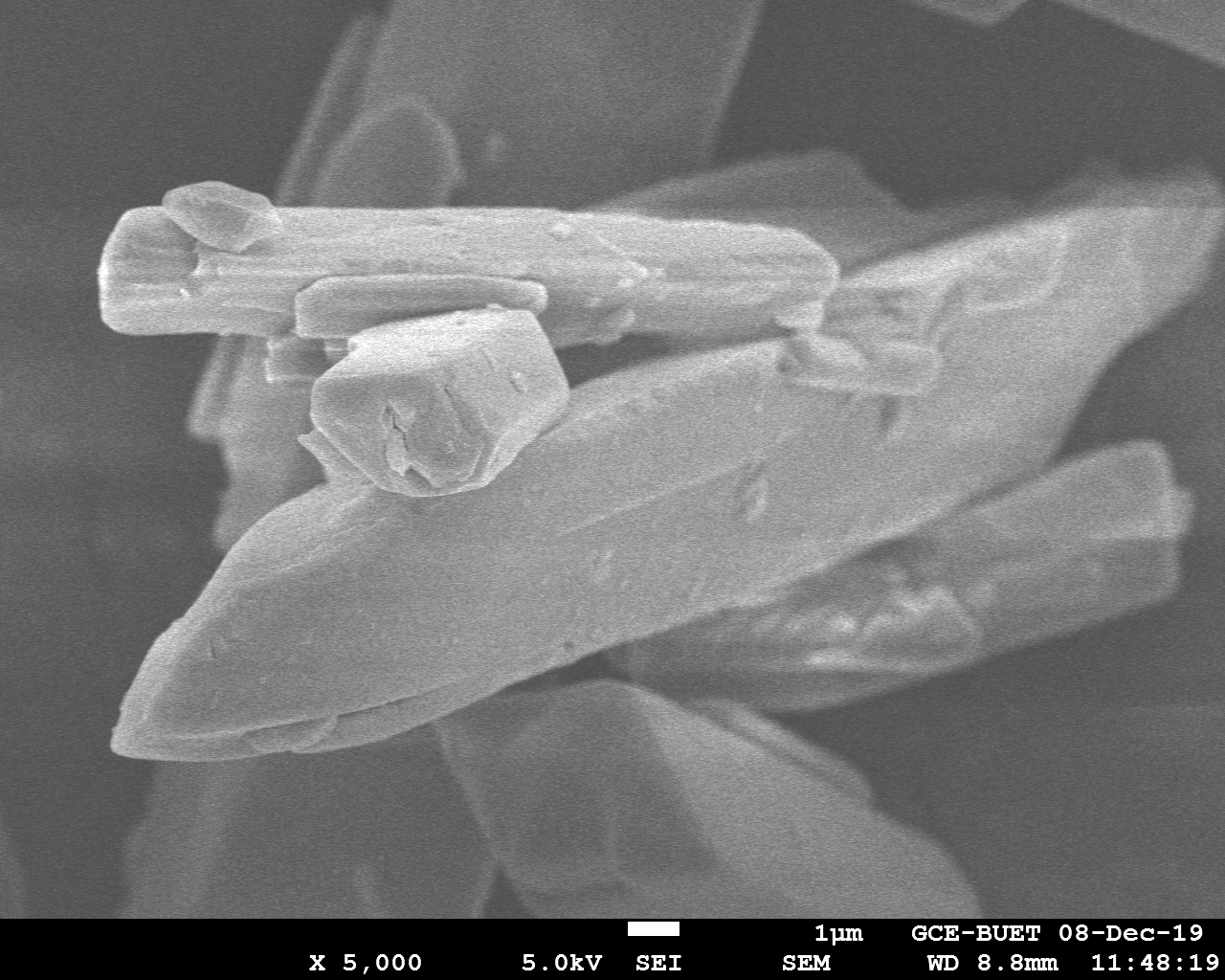

Supplement: S1 File — S1 Figure. Standard calibration curve of pure ampicillin in distilled water at 216 nm. S1 Appendix. UV-visible Spectroscopy Data. S2 Appendix. FTIR Data. S3 Appendix. DLS and Zeta Potential Data. S4 Appendix. SEM Data. S5 Appendix. EDX Data. S6 Appendix. TGA Data. S7 Appendix. AgNP-ampicillin Synthesis Reaction. S8 Appendix. Microbiological Study Data. S9 Appendix. Molecular Docking Data. S10 Appendix. Cytotoxicity Assay Procedure. (ZIP) [file pone.0331669.s001.zip › Supporting Informations/S4_Appendix (SEM Data)/Ampicillin.jpg]

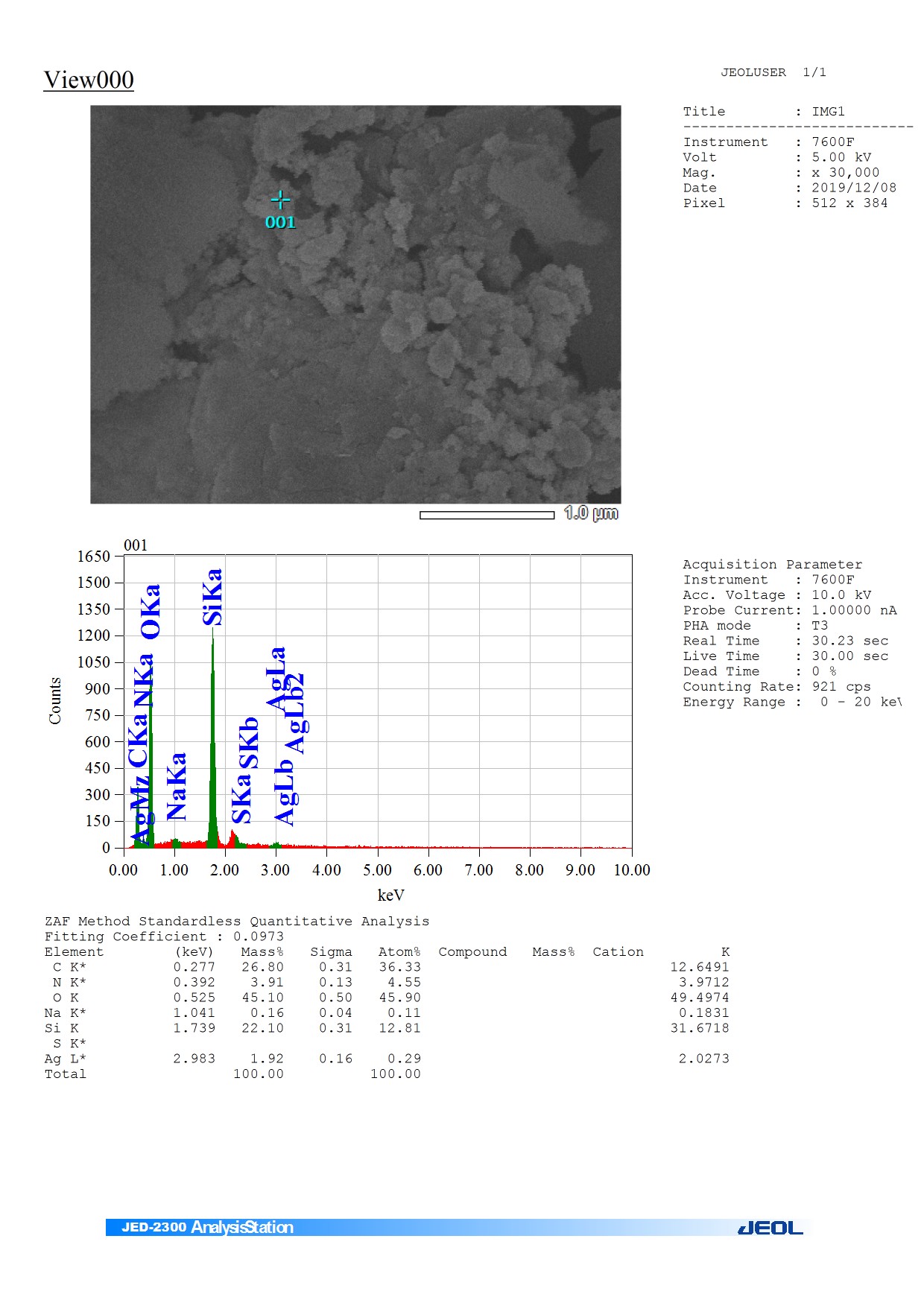

Supplement: S1 File — S1 Figure. Standard calibration curve of pure ampicillin in distilled water at 216 nm. S1 Appendix. UV-visible Spectroscopy Data. S2 Appendix. FTIR Data. S3 Appendix. DLS and Zeta Potential Data. S4 Appendix. SEM Data. S5 Appendix. EDX Data. S6 Appendix. TGA Data. S7 Appendix. AgNP-ampicillin Synthesis Reaction. S8 Appendix. Microbiological Study Data. S9 Appendix. Molecular Docking Data. S10 Appendix. Cytotoxicity Assay Procedure. (ZIP) [file pone.0331669.s001.zip › Supporting Informations/S5_Appendix (EDX Data)/AgNP-amp (Point-1).jpg]

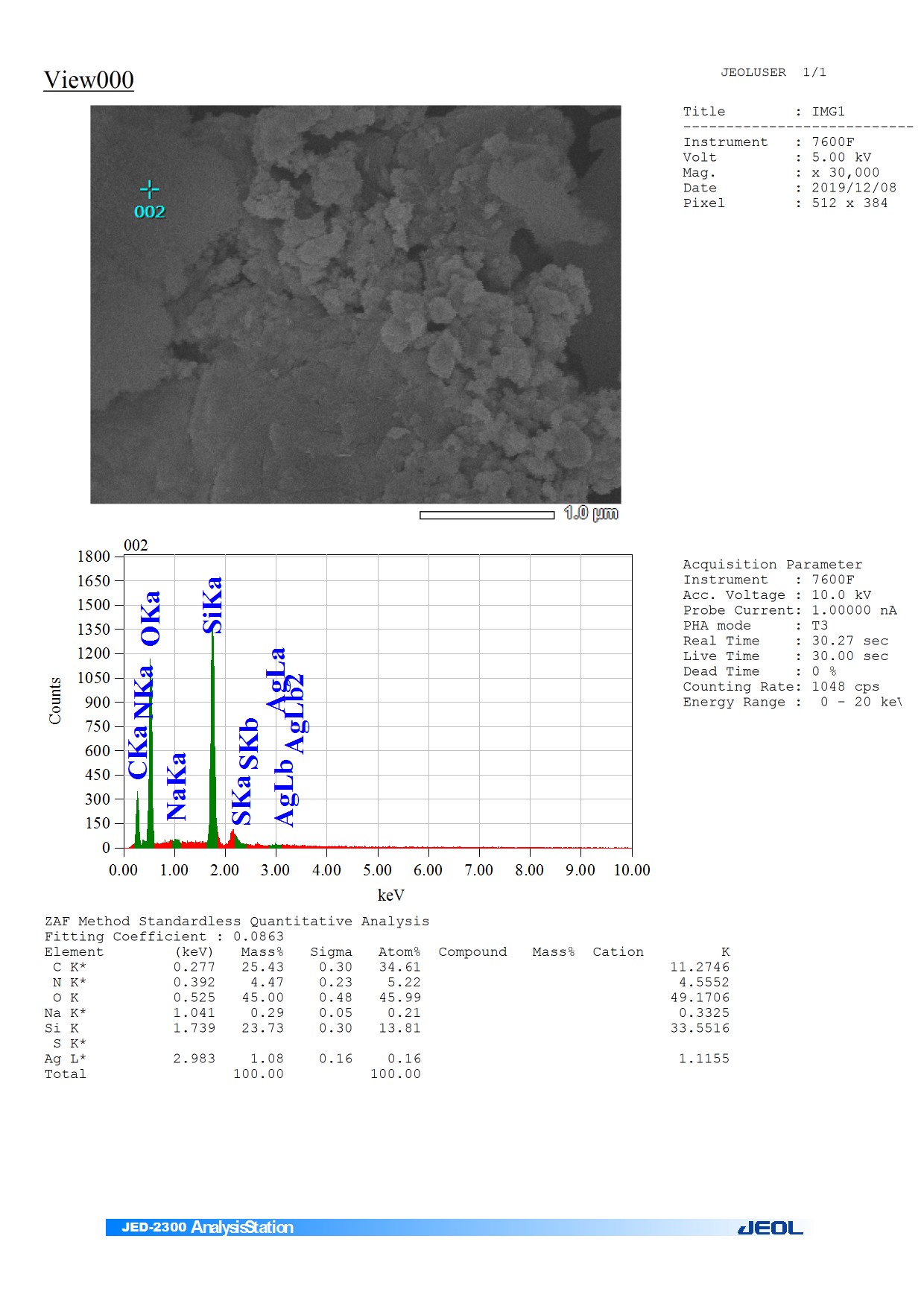

Supplement: S1 File — S1 Figure. Standard calibration curve of pure ampicillin in distilled water at 216 nm. S1 Appendix. UV-visible Spectroscopy Data. S2 Appendix. FTIR Data. S3 Appendix. DLS and Zeta Potential Data. S4 Appendix. SEM Data. S5 Appendix. EDX Data. S6 Appendix. TGA Data. S7 Appendix. AgNP-ampicillin Synthesis Reaction. S8 Appendix. Microbiological Study Data. S9 Appendix. Molecular Docking Data. S10 Appendix. Cytotoxicity Assay Procedure. (ZIP) [file pone.0331669.s001.zip › Supporting Informations/S5_Appendix (EDX Data)/AgNP-amp (Point-2).jpg]

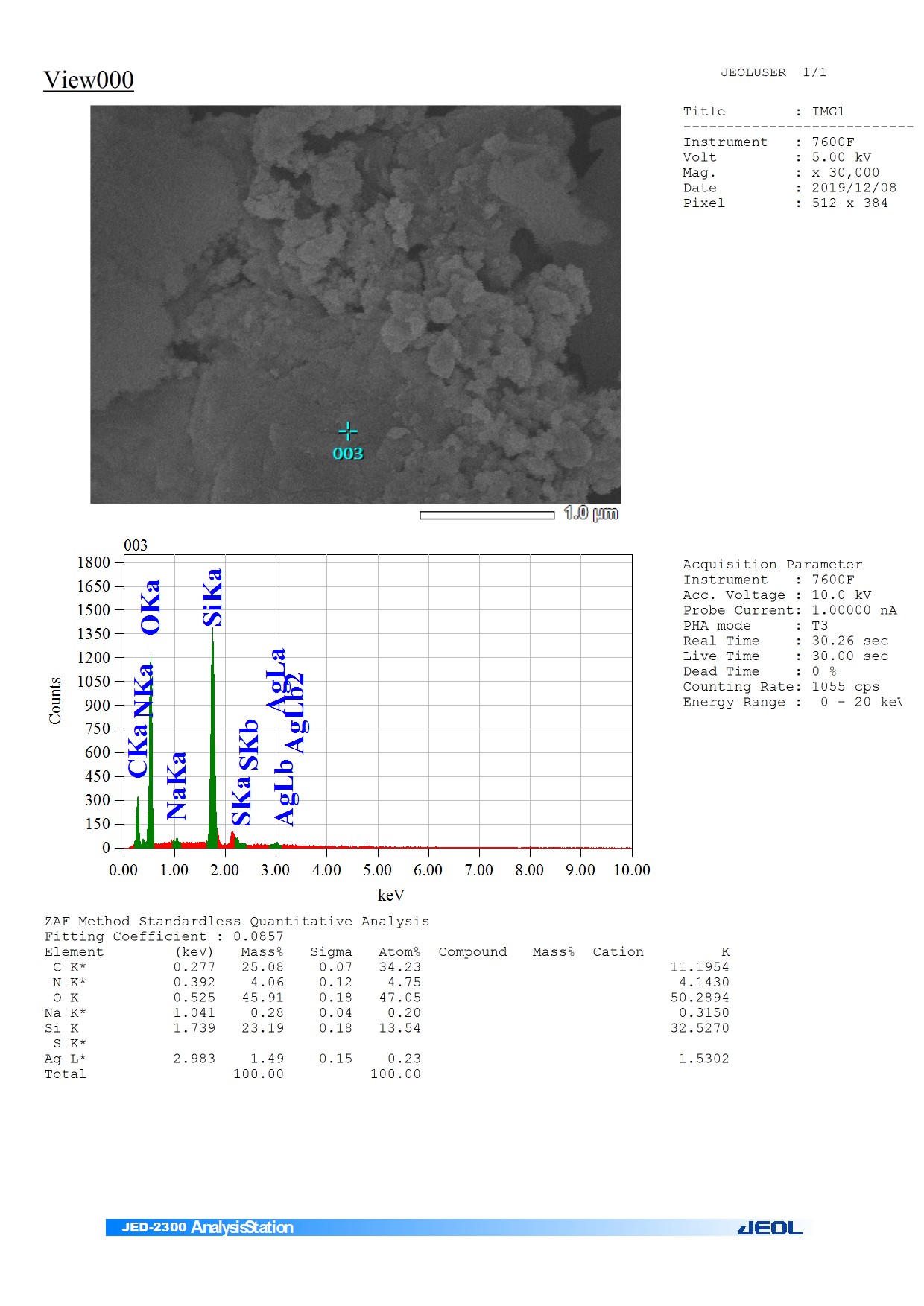

Supplement: S1 File — S1 Figure. Standard calibration curve of pure ampicillin in distilled water at 216 nm. S1 Appendix. UV-visible Spectroscopy Data. S2 Appendix. FTIR Data. S3 Appendix. DLS and Zeta Potential Data. S4 Appendix. SEM Data. S5 Appendix. EDX Data. S6 Appendix. TGA Data. S7 Appendix. AgNP-ampicillin Synthesis Reaction. S8 Appendix. Microbiological Study Data. S9 Appendix. Molecular Docking Data. S10 Appendix. Cytotoxicity Assay Procedure. (ZIP) [file pone.0331669.s001.zip › Supporting Informations/S5_Appendix (EDX Data)/AgNP-amp (Point-3).jpg]

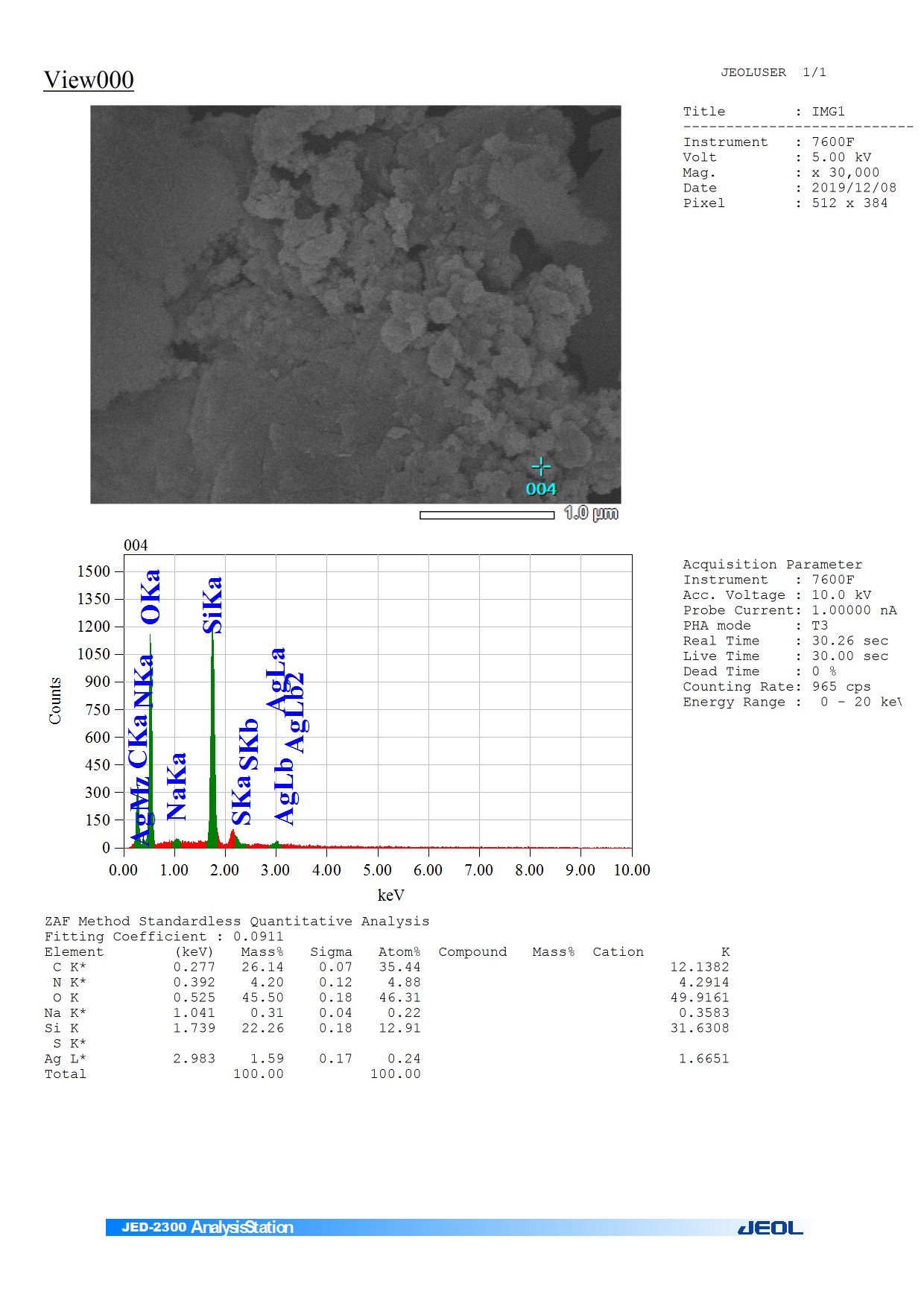

Supplement: S1 File — S1 Figure. Standard calibration curve of pure ampicillin in distilled water at 216 nm. S1 Appendix. UV-visible Spectroscopy Data. S2 Appendix. FTIR Data. S3 Appendix. DLS and Zeta Potential Data. S4 Appendix. SEM Data. S5 Appendix. EDX Data. S6 Appendix. TGA Data. S7 Appendix. AgNP-ampicillin Synthesis Reaction. S8 Appendix. Microbiological Study Data. S9 Appendix. Molecular Docking Data. S10 Appendix. Cytotoxicity Assay Procedure. (ZIP) [file pone.0331669.s001.zip › Supporting Informations/S5_Appendix (EDX Data)/AgNP-amp (Point-4).jpg]

# Thermal Analysis Result, Centre for Advanced Research in Sciences, University of Dhaka

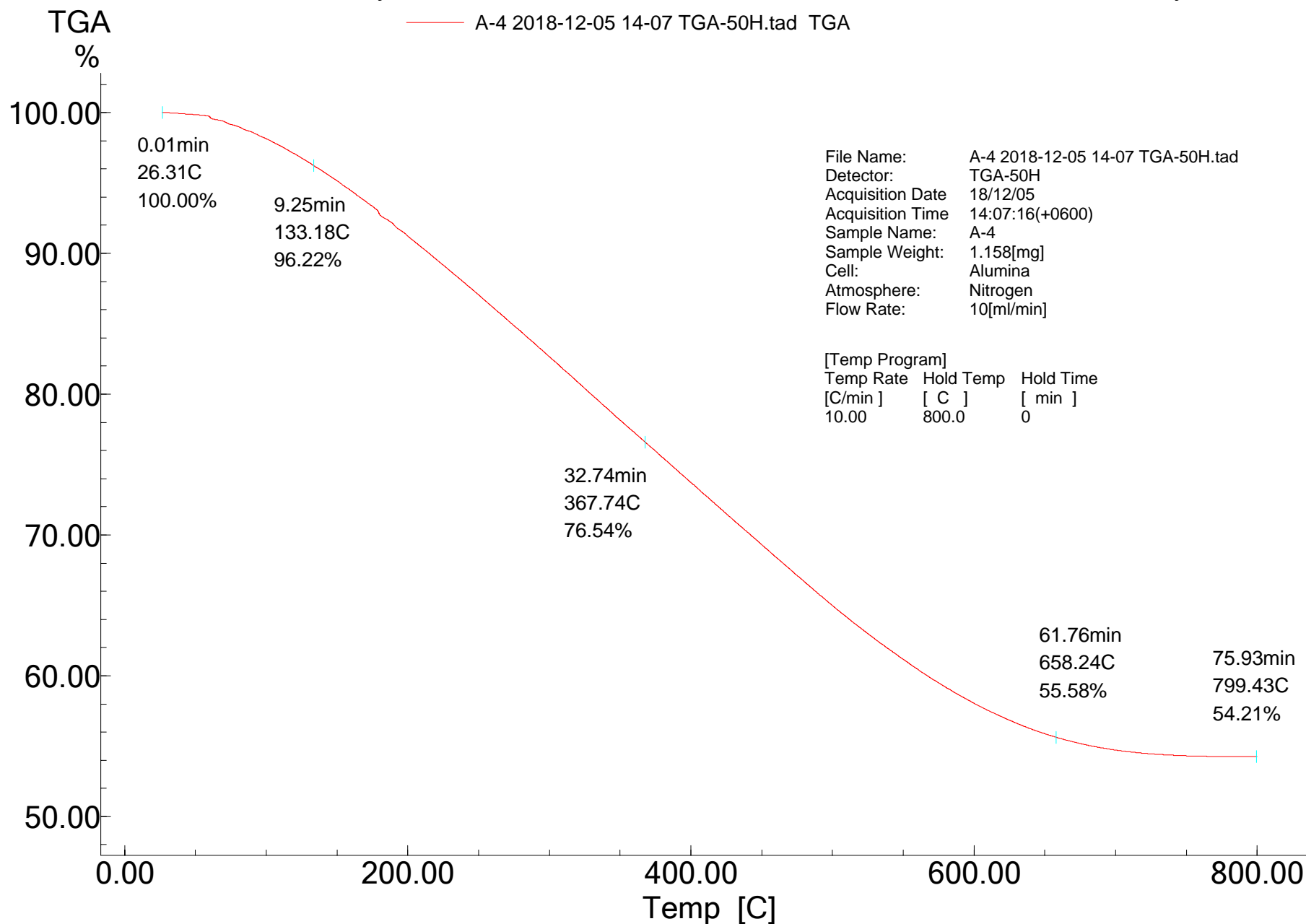

Supplement: S1 File — S1 Figure. Standard calibration curve of pure ampicillin in distilled water at 216 nm. S1 Appendix. UV-visible Spectroscopy Data. S2 Appendix. FTIR Data. S3 Appendix. DLS and Zeta Potential Data. S4 Appendix. SEM Data. S5 Appendix. EDX Data. S6 Appendix. TGA Data. S7 Appendix. AgNP-ampicillin Synthesis Reaction. S8 Appendix. Microbiological Study Data. S9 Appendix. Molecular Docking Data. S10 Appendix. Cytotoxicity Assay Procedure. (ZIP) [file pone.0331669.s001.zip › Supporting Informations/S6_Appendix (TGA Data)/AgNP@SiO2-NH2.pdf]

# Thermal Analysis Result, Centre for Advanced Research in Sciences, University of Dhaka

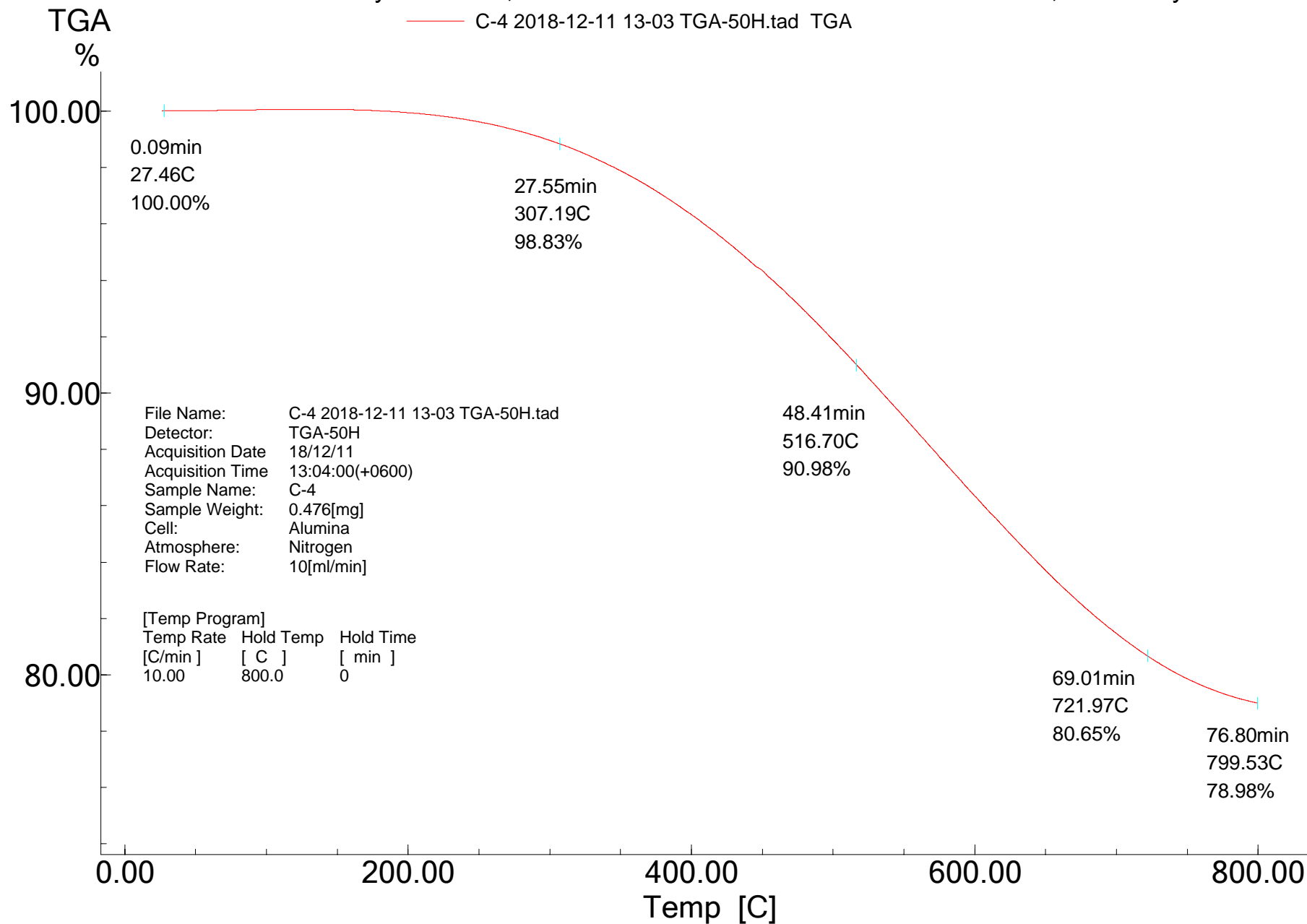

Supplement: S1 File — S1 Figure. Standard calibration curve of pure ampicillin in distilled water at 216 nm. S1 Appendix. UV-visible Spectroscopy Data. S2 Appendix. FTIR Data. S3 Appendix. DLS and Zeta Potential Data. S4 Appendix. SEM Data. S5 Appendix. EDX Data. S6 Appendix. TGA Data. S7 Appendix. AgNP-ampicillin Synthesis Reaction. S8 Appendix. Microbiological Study Data. S9 Appendix. Molecular Docking Data. S10 Appendix. Cytotoxicity Assay Procedure. (ZIP) [file pone.0331669.s001.zip › Supporting Informations/S6_Appendix (TGA Data)/AgNP@SiO2.pdf]

# Thermal Analysis Result, Centre for Advanced Research in Sciences, University of Dhaka

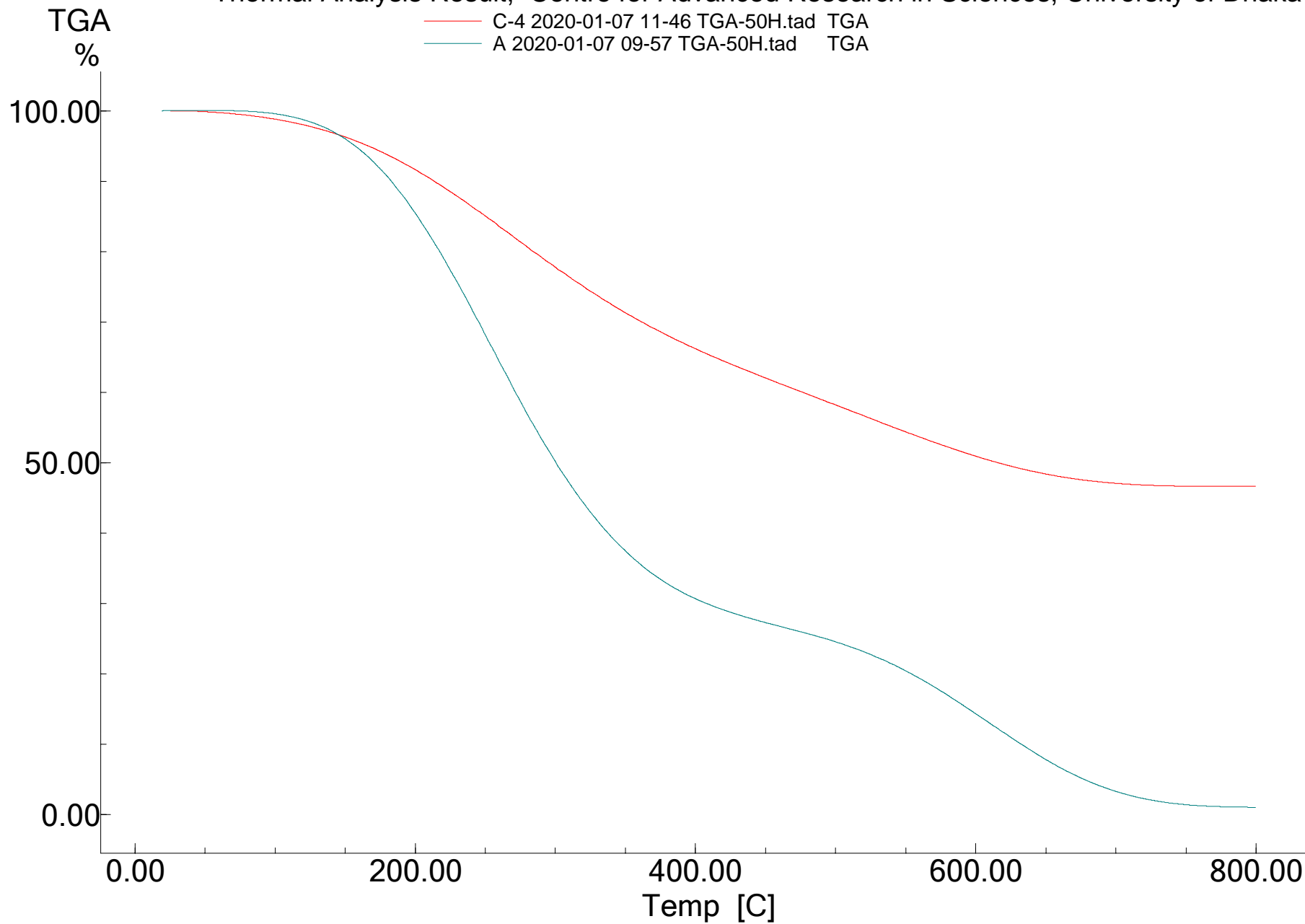

Supplement: S1 File — S1 Figure. Standard calibration curve of pure ampicillin in distilled water at 216 nm. S1 Appendix. UV-visible Spectroscopy Data. S2 Appendix. FTIR Data. S3 Appendix. DLS and Zeta Potential Data. S4 Appendix. SEM Data. S5 Appendix. EDX Data. S6 Appendix. TGA Data. S7 Appendix. AgNP-ampicillin Synthesis Reaction. S8 Appendix. Microbiological Study Data. S9 Appendix. Molecular Docking Data. S10 Appendix. Cytotoxicity Assay Procedure. (ZIP) [file pone.0331669.s001.zip › Supporting Informations/S6_Appendix (TGA Data)/Overlay of Ampicillin & AgNP-ampicillin.pdf]
