# Supplementary material for: Synergistic antibacterial action of AgNP-ampicillin conjugates: Evading β-lactamase degradation in ampicillin-resistant clinical isolates
Source: PLoS One. 2025 Sep 9;20(9):e0331669. doi: 10.1371/journal.pone.0331669 (PMC12419620; doi:10.1371/journal.pone.0331669)
Supplement: Attachment — Submitted filename: Review_2_plos.pdf [file pone.0331669.s002.pdf]

Dear Editor,

This study tackles a critical challenge in combating methicillin-resistant bacteria and proposes an innovative solution through the use of ampicillin-conjugated silver nanoparticles (AgNPs). However, the article requires substantial revisions to enhance scientific clarity and improve readability. A thorough rewrite is recommended.

Thanks & Regards.

General notes:

- Mechanism: The study does not discuss *how* the AgNP-ampicillin conjugate exerts its enhanced antimicrobial activity. Potential mechanisms, such as ROS generation, bacterial membrane disruption, or interference with resistance pathways, are not explored. Understanding the mechanism can provide insights into optimizing this strategy and addressing resistance.
- Toxicity: The study does not address the potential cytotoxicity of the AgNP-ampicillin conjugate toward human cells or environmental safety concerns. For clinical application, assessing the safety profile is critical. Nanoparticles are known to have cytotoxic effects, and their use requires a careful balance between efficacy and biocompatibility.
- In vivo: The study is entirely in vitro. Testing the conjugates in animal models or cell line testing would strengthen its translational potential. The in vivo environment differs significantly from in vitro setups, and efficacy in a biological system would better predict clinical success.
- Stability: Long-term stability of the AgNP-ampicillin conjugate under various storage conditions is not discussed. Stability is essential for practical use and commercialization.

Review:

1. **Title** can be improved, should be concise

   Increased Efficacy of Ampicillin Conjugated with Silver Nanoparticle Against Methicillin-Resistant Clinical Isolates

Synthesis and Microbiological Characterization of Silver Nanoparticle Conjugated Ampicillin

2. **Abstract:**

- The abstract is slightly lengthy for a typical journal format and could be more concise.
- Could briefly acknowledge the need for further studies (e.g., in vivo efficacy or toxicity testing).
- Adding numerical data's can be avoided in abstract for clarity (eg. (15, 14, 13.5, 13 mm))

3. **Background**

Overloading of Information:

- The section contains extensive detail about multiple nanoparticles (e.g., gold, titanium dioxide, copper, etc.), which may detract from the focus on silver nanoparticles (AgNPs) and ampicillin.
- Redundant statements about antibiotic resistance mechanisms could be condensed.

Lack of Flow in Transition:

- The shift from the problem of resistance to the use of AgNPs and then to antibiotic conjugates could be made smoother for better readability.

Mechanism of Action for AgNPs and Ampicillin Conjugates:

- While AgNPs' antimicrobial activity is discussed, a more focused discussion on the synergy between AgNPs and ampicillin is missing.

Objective and Novelty Statement:

- The objective and novelty of the study are included at the end but are somewhat generic and lack a concise statement emphasizing what sets this study apart from previous research.

Mechanistic Insights:

- The mention of “developing the mechanism of synthesis and antibacterial action” lacks specificity and clarity. This could be better explained in light of the study’s scope.

4. **Materials**

- Indicate the grade or purity of chemicals (e.g., "analytical grade," "99.9% pure") where applicable. This is particularly important for reproducibility.

5. **Methods**

- Ensure consistent use of units (e.g., "ml" vs. "mL"). In scientific writing, "mL" is the standard.
- Briefly describe the role of key reagents (e.g., silver nitrate as the precursor, trisodium citrate as the stabilizing agent, sodium borohydride as the reducing agent, and hydrogen peroxide as an oxidizing agent).
- Specify the temperature during centrifugation
- For reproducibility, add notes on how the synthesized nanoparticles were stored after purification (e.g., suspension in ethanol or drying conditions).
- Use consistent notation for units (e.g., "µl" vs. "µL"). The standard is "µL."
- Mention that tetraethyl orthosilicate (TEOS) is the silica precursor for readers unfamiliar with the process.
- Briefly describe the role of ammonia as a catalyst for silica shell formation.
- Include details on the total reaction time overnight (e.g., "12 hours") if known.
- Specify the temperature during centrifugation (e.g., room temperature or a specific value if critical).
- State how the synthesized nanoparticles were stored after purification.
- **Concentration of APTES**: While the volume of APTES is mentioned (100 µL), its concentration or molarity in the reaction mixture is not provided. This makes reproducibility challenging.
- **Sonication Time and Conditions**: Sonication is mentioned, but the intensity, frequency, or power settings are not specified. These parameters can affect the outcome.
- **Purification Clarity**: The washing and centrifugation steps lack details about the solvent used for washing after ethanol or the number of purification cycles.

- Add a note on the ratio of EDC/NHS to amine groups for optimal conjugation.
- Include a method to quantify unreacted ampicillin in the supernatant to confirm the efficiency of conjugation.
- **UV-Vis Spectroscopy**: Details about the wavelength range and resolution for absorbance measurements are missing.
- **DLS and Zeta Potential**: The solvent used for DLS measurements is not mentioned. The influence of different media (water vs ethanol) on zeta potential should be clarified.
- **SEM Analysis**: Preparation of samples for SEM (e.g., coating, freeze-drying process) is missing.
- **FTIR Analysis**: Conditions such as sample preparation method (e.g., pelletization or direct smear), baseline correction, and software used for analysis are not mentioned.
- **TGA**: The initial mass of the sample for TGA is not mentioned, nor are the specific nitrogen flow rate and calibration standards.

Evaluation of Antibacterial Activity

- **Control Samples**: Although the antibacterial activity of several compounds (AgNO3, AgNPs, AgNP-ampicillin, and pure ampicillin) is being tested, the types and concentrations of control agents (such as a positive control, e.g., a known antibiotic or solvent control) are not mentioned. This is critical for validating the experiment.
- **Experimental Design**: The number of replicates for each sample and control (e.g., biological replicates, technical replicates) is not mentioned. This should be clearly stated to ensure statistical relevance.

Disk Diffusion Test

- **Preparation of the Agar**: While the method describes dissolving MHA powder in distilled water, it would be helpful to provide the pH adjustment of the medium (if done), as pH can affect microbial growth and antimicrobial activity.
- **Organism Transfer Method**: The description of transferring organisms from a subculture into sterile media for inoculation could be clearer. It would be useful to mention if the bacterial suspension was standardized (e.g., using a

spectrophotometer to match an optical density at 600 nm) to ensure consistent inoculum density.

- **Filter Disk Handling**: The drying process for the filter disks soaked with the test samples could be clarified. It is important to mention how long the disks should be dried to ensure uniform distribution of the test sample on the disk.
- **Incubation Conditions**: The 20-hour incubation period at 37°C seems lengthy; it could be beneficial to clarify if this is optimal or if it could be shortened.

MIC, MBC, and MPC Assays

- **Sample Concentrations**: The concentration of the test samples used in the MIC, MBC, and MPC assays (e.g., 500 µg of test sample) is provided, but it's unclear if this concentration is consistent across different assays or if there were variations based on sample type.
- **Dilution Series**: For the MIC and MBC assays, the dilution process is described as a 1:2 dilution series, but the final concentrations of the test solutions are not clear. The rationale behind the starting concentration (500 µg/ml) and how it relates to the bacterial strains' expected sensitivity could be expanded.
- **Sterility Control**: The sterility controls are mentioned but need further explanation, such as whether they contain only the media or if a bacterial inoculum is added without the test compound.
- **MPC Test**: The MPC assay is introduced but lacks details on how it specifically differs from the MIC and MBC tests in terms of experimental conditions or how bacterial mutation rates are monitored.
- **Timing of Measurements**: For both MIC and MBC, the incubation time and temperature are clearly provided (37°C for 16–20 hours). However, the experiment would benefit from more specific recommendations on time points for monitoring. Ensure that timing and temperature for incubation are optimized and explain how these parameters were determined.

MBIC and MBEC assay

- **Consistency in terminology**: You mention the use of "serial dilutions" (1:2 dilution) in both MBIC and MBEC assays but do not specify how the dilution steps are implemented in terms of volume (e.g., how much of the initial solution is taken and diluted). This should be clarified for both assays.
- **Test tube setup for MBEC assay**: In the MBEC section, the text mentions preparing 2 ml of sterile broth in screw-cap test tubes and then adding bacterial subcultures (100 µl of bacteria in 9.9 ml of media) and then adding 20 µl per test tube. This is confusing because it seems like a mix of volume for inoculum and sample additions. It would be better to clearly outline the procedure for both inoculation and treatment steps to avoid ambiguity.
- **Crystal violet concentration**: The concentration of crystal violet solution is described consistently as 0.1%, but it would be helpful to note the volume of crystal violet used for staining, especially in the MBEC assay where a different number of test tubes might be involved.
- **Absorbance measurements**: It's mentioned that the absorbance is measured at 590 nm for both MBIC and MBEC assays, but it would be helpful to elaborate on how the absorbance readings correlate with biofilm formation or eradication (e.g., at what level absorbance represents significant inhibition or eradication). Additionally, how many replicates are used for these measurements? It's important to clarify that these readings are averaged over multiple replicates for consistency and reliability of results.

In the **MBIC assay**, you mention a 24-hour incubation at 37°C for biofilm formation, but the **MBEC assay** mentions a subsequent 4-hour incubation after the addition of test samples. The transition between these incubation periods should be clarified. For example, is the 4-hour incubation for biofilm eradication after the 24-hour incubation for biofilm formation, or does it happen simultaneously?

- In several parts of your protocol, there is a bit of redundancy (for example, explaining the process of preparing MHB and MHA, followed by autoclaving). Consider streamlining sections where the procedure is repeated, especially when it pertains to preparing media, autoclaving, and adding bacteria. You can reference these steps

once and then continue with the specific experimental steps for MBIC and MBEC assays.

- In the **MBEC assay**, you mention that after a 4-hour incubation, biofilms are stained with crystal violet. However, in the description, it is unclear whether the stained biofilm is washed again or if any further steps are taken before measuring absorbance. Clarifying these steps could make the procedure more robust. Typically, after biofilm eradication, the wells/plates are washed before crystal violet staining to remove any unbound dye.
- While you mention the inclusion of growth and sterility controls in both assays, it would be useful to briefly describe how the controls are set up. For example, are the sterility controls prepared with just media and no bacteria, and how are the growth controls set up (e.g., samples with bacterial inoculum but no treatment)?

Time-kill kinetic assay

Clarity on Time Intervals:

- The text mentions "samples were taken at intervals" but does not specify what those intervals are. Are the samples taken at specific time points such as 1, 2, 4, and 8 hours? It is crucial to define the sampling intervals clearly in the methodology so readers understand the time course of the experiment.

Clarification on the Use of Growth and Sterility Controls:

- In the description, growth control and sterility controls are prepared with 2 ml of sterile broth, but it is not clear how these controls are handled in terms of sample preparation. You could clarify:
    - For the **growth control**, it would be important to add bacteria without any treatment to see if the bacteria grow in the absence of AgNO3, AgNPs, AgNP-ampicillin, or ampicillin.
    - For **sterility control**, this should include sterile broth with no bacteria, ensuring that no contamination occurs.

- Also, you could mention whether these controls are incubated under the same conditions as the experimental samples.

Preparation of Bacterial Suspension:

- The step where a bacterial subculture is diluted and added to the vials (20 µl per vial) needs more detail. Specifically:
    - **How was the bacterial subculture prepared?** Was it grown in a liquid medium beforehand? How was the initial bacterial concentration measured?
    - **Why 20 µl per vial?** Is this an optimized amount for the specific bacterial strain being used? Explaining this would make the protocol clearer.

Bacterial Viability Assessment:

- The procedure describes spreading **10 µl of the incubated suspension on agar plates**. It's worth clarifying if the entire 10 µl is spread on the plate in one go or if it is distributed in separate drops. If it is spread in drops, you mention "10 µl/drop × 5 drops/inoculum," but it might be clearer to state something like, "The 10 µl was used to create five 2 µl drops, each dropped separately on the plate."
- The method specifies incubating the plates at **37°C for 16 hours**. This is a standard practice, but you might want to specify the reason for this incubation period (i.e., how it ensures proper growth of the bacteria for colony counting).

Time-Kill Assay Analysis:

- The **log10(CFU/ml) reduction** is a standard way of measuring bactericidal activity, but it would be useful to clarify how exactly the readings are taken:
    - Are the colonies counted manually, or is an automated system used?
    - How are the counts converted into log10 values?
    - Is the 3 log10(CFU/ml) reduction considered for all bacterial strains, or does it vary depending on the strain's susceptibility?

- When referring to the test samples (e.g., $AgNO_3$, AgNPs, AgNP-ampicillin, and ampicillin), it would be helpful to provide more detail about the preparation of these solutions:
    - **How are these solutions prepared?** Is there any dilution factor considered? Are these solutions prepared fresh or stored?
    - For example, you mention "500 µg of test samples," but it would be clearer to specify if this is per ml or if it's the total amount added to the vials.

- The protocol mentions **500 µg of test samples**, but it does not specify if this concentration is being used for each bacterial strain or if it is a standard concentration for all strains. Including this information would help standardize the assay, ensuring reproducibility across different bacterial isolates.

- The preparation of **Mueller-Hinton Broth (MHB)** is repeated in your text. Instead of detailing this twice (once for the vials and once for dilution in plates), you could consolidate the media preparation steps for clarity.

- In one section, the protocol states, "1 ml of sterile media and 1 ml of solutions containing 500 µg of test samples were placed in screw cap glass vials." But it's unclear whether this is the total volume of the solution in each vial or if it's intended for different time points. It might be better to clarify that each vial receives 500 µg/ml of test sample in 2 ml of final solution.
- It may also be worth specifying the **final concentration of the test sample in the bacterial suspension**. Does the final concentration of $AgNO_3$, AgNPs, AgNP-ampicillin, and ampicillin stay constant throughout the assay?

- The sentence "A concentration or time dependent effect of $AgNO_3$, AgNPs, AgNP-ampicillin and pure ampicillin on the strains of bacteria were measured by this assay" could be reworded for clarity.

FIC Index Formula Clarification:

- The formula for the **FIC Index** is stated as:
    - **FIC Index = FICA + FICB = (A/MICA) + (B/MICB)**

- It is important to clarify that:
  - **FICA** is the fractional inhibitory concentration of **ampicillin (A)** in the combination.
  - **FICB** is the fractional inhibitory concentration of **AgNP (B)** in the combination.
- The notation is correct, but it could be reworded for better understanding.

Suggested rewording:

- **FIC Index = FICA + FICB**
  - Where:
    - **FICA** = A / MICA (Concentration of ampicillin in the combination divided by the MIC of ampicillin alone)
    - **FICB** = B / MICB (Concentration of AgNPs in the combination divided by the MIC of AgNPs alone)

- The **interpretation of FIC values** is well-explained, but the definition of the ranges could be more precise in terms of explaining **synergy** and **antagonism**:
  - **FIC ≤ 0.5:** Synergistic effect. This means the combination of ampicillin and AgNPs is more effective than either of them alone, and the inhibition of bacterial growth is enhanced.
  - **0.5 < FIC ≤ 1:** Additive effect. This means the combination of ampicillin and AgNPs has an effect equal to or similar to the sum of their individual effects, but no enhancement.
  - **1 < FIC ≤ 2:** Indifference effect. This means the combination does not show a significant improvement over the individual effects, and their interaction is considered neutral.
  - **FIC > 2:** Antagonistic effect. This means the combination of ampicillin and AgNPs is less effective than either of them alone, indicating that one compound may be reducing the effectiveness of the other.

These definitions of the effect types (synergistic, additive, indifference, and antagonistic) should be emphasized in the text for clarity.

- The sentence, "The FIC index value considers the antibiotic combination that results in the largest deviation from the individual antibiotic's MIC" could be clarified further:
    - The FIC Index actually calculates the sum of the ratios of the combination concentration to the individual MICs, rather than focusing on "deviation." A clearer way to state this could be: "The FIC index value quantifies the interaction between two compounds by summing the ratios of each compound's concentration in the combination to its individual MIC."

- The method for determining **MIC** (Minimum Inhibitory Concentration) should be briefly mentioned again in this section for context. It's useful to remind the reader that the MIC values for ampicillin and AgNPs should be determined separately before calculating the FIC index.

- In the sentence, "The FIC index value considers the antibiotic combination that results in the largest deviation from the individual antibiotic's MIC," consider revising for better clarity.

- Including a brief mention of the concentration ranges used for ampicillin and AgNPs when determining the MIC values will add completeness to the methodology.

RESULTS & DISCUSSIONS

Chemical Characterization of AgNPs, Ampicillin, and AgNP-ampicillin:

- **"380 cm-1 to 440 cm-1"**: The wavelength units for light absorption are generally given in **nm** (nanometers) rather than cm-1. If you're referring to the range of absorption for the silver nanoparticles' surface plasmon resonance, it should be **380–440 nm**, not cm-1.
- **"TGA curve (Figure 1, C) indicated AgNP formation (47)"**: The TGA data would likely indicate mass loss, not necessarily the "formation" of AgNPs. You might want to

rephrase this, perhaps something like: "The TGA curve (Figure 1, C) shows characteristic mass loss patterns consistent with the formation of AgNPs."

- **"Z-Average is -36.7 mV indicates better stability profile as it is more negative than -30 mV (48)"**: This sentence is grammatically unclear. It should be rephrased for clarity: "The Z-Average of -36.7 mV indicates a better stability profile, as values more negative than -30 mV suggest enhanced stability ."
- **"The SEM image (Figure 1, F) of the synthesized AgNPs also confirms the formation of nanoparticles in ranges similar to those obtained by DLS"**: It would be more accurate to say that SEM provides **visual confirmation** of the size and morphology, whereas DLS gives a **statistical distribution** of particle sizes. Consider clarifying this distinction.

2. Characterization of Silica-Coated and Amine-Functionalized AgNPs:

- **"Increase degradation in TGA (Figure 3, A) was observed due to amine functionalization of AgNPs"**: The term "increase degradation" is unclear. You likely mean an **increase in weight loss** or **changes in thermal stability**. Rephrase this for clarity, e.g., "Increased weight loss in the TGA curve (Figure 3, A) was observed due to the amine functionalization of AgNPs."

3. Characterization of AgNP-ampicillin Conjugate:

- **"In the FTIR data of AgNP-ampicillin (Figure 4, B), AgNP-ampicillin shows a robust broad band in the range of 3300 to 3500 cm-1 for the N-H stretch"**: Again, the unit for this range seems inconsistent with common FTIR measurements (usually in **cm^{-1}** rather than **nm**). Verify that you're using the correct units.
- **"The peak at 1113 cm−1 in AgNP-ampicillin confirms the stretching vibration of the silyl ether group, which supports the conjugation between TEOS and APTES in amine-functionalized AgNPs (50)"**: This is a good explanation, but you should ensure that the reference to TEOS and APTES is clear for readers unfamiliar with these reagents. Briefly explain these abbreviations or add a reference to them.

4. Yield Calculation:

- **"Theoretical amount of AgNP-ampicillin present in the conjugated product was equivalent to 12 milligrams of pure ampicillin"**: This could be confusing, as it's not clear how you arrived at this estimate. Consider adding a brief explanation of the calculation for clarity.

5. Functional Characterization of AgNPs, Ampicillin, and AgNP-ampicillin:

- **Disk Diffusion Assay**: You mentioned "zone of inhibitions" which should be corrected to **zone of inhibition.**
- **"The MIC, MBC, and MPC values suggested increased antibacterial activity of AgNP-ampicillin in comparison to pure ampicillin"**: This is a good comparison, but it's important to discuss why AgNP-ampicillin is more effective in detail, possibly referring to the enhanced stability or synergy between AgNPs and ampicillin.

6. MBIC and MBEC Assay:

- **"MBEC values stipulated a significant increase in the anti-biofilm activity of AgNP-ampicillin in contrast to pure ampicillin"**: Mention how AgNP-ampicillin's improved anti-biofilm activity could be attributed to the nanoparticles' interaction with biofilms or their ability to penetrate biofilm matrices.

7. Time-Kill Kinetic Assay:

- **"Slightly rapid antibacterial activity of AgNP-ampicillin was observed when compared with pure ampicillin within 8 hours"**: "Slightly rapid" is somewhat vague. Instead, quantify the improvement in terms of **log reductions** in bacterial counts, and explicitly state how much faster AgNP-ampicillin works compared to ampicillin alone.

Conclusion

- The phrase "multiple treatments using broad-spectrum antibiotics" can be rephrased to specify what you mean by "multiple treatments." Do you mean "repeated treatments" or "the use of multiple broad-spectrum antibiotics"?

Scientific Detail:

- The sentence "The study reveals the quantity of ampicillin molecules attached to each AgNP" could be more specific. If possible, provide details on how much ampicillin was attached or the method used for quantification.
- "However, increasing the number of ampicillin molecules attached to each AgNP will reduce the toxicity associated with AgNPs while increasing the bioavailability of ampicillin." This sentence seems to imply that attaching more ampicillin reduces toxicity. While this may be the case, it's not immediately clear why this would be true. Perhaps provide a clearer explanation or reference supporting this claim.

Sentence Structure:

- The phrase "The study also demonstrated the synthesis mechanism of AgNP-ampicillin conjugates" could be more explicit. What exactly was demonstrated about the synthesis mechanism?
- "The AgNP-ampicillin conjugates showed a significant increase in the bactericidal activity, which might be the consequence of increased permeability to the bacterial cell wall and reduced beta-lactamase binding with ampicillin." This sentence could benefit from a clearer transition and a more direct connection between the observed effects and the proposed mechanisms.

Flow and Conclusion:

- The last sentence could be slightly rephrased for a smoother flow.
