# Supplementary material for: Synergistic antibacterial action of AgNP-ampicillin conjugates: Evading β-lactamase degradation in ampicillin-resistant clinical isolates
Source: PLoS One. 2025 Sep 9;20(9):e0331669. doi: 10.1371/journal.pone.0331669 (PMC12419620; doi:10.1371/journal.pone.0331669)
Supplement: Attachment — Submitted filename: Response to Reviewers.docx [file pone.0331669.s004.docx]

**Manuscript Title**: Increased Efficacy of Ampicillin Conjugated with Silver Nanoparticle Against Methicillin-Resistant Clinical Isolates

**Manuscript ID**: PONE-D-24-53049

**Dear Academic Editor and Reviewers,**

We sincerely thank you for your thoughtful and constructive feedback on our manuscript. Your comments and suggestions have been invaluable in improving the quality and clarity of our work. We have carefully addressed all the points raised and revised the manuscript accordingly. Below, we provide detailed responses to each of the reviewers' and editor's comments. For ease of reference, we have restated the comments in **bold** and provided our responses below each. All changes made to the manuscript are highlighted in the revised version.

**Responses to Academic Editor's Comments**

**1. Please ensure that your manuscript meets PLOS ONE's style requirements, including those for file naming.**

**Response:** We have revised the manuscript to adhere to PLOS ONE's style requirements. Additionally, all file names have been updated to comply with the journal's standards.

**2. Funding information should not appear in any section of the manuscript except for the Funding Statement section of the online submission form.**

**Response:** All funding-related text has been removed from the manuscript body and included only in the Funding Statement section, as per journal guidelines.

**3. Please state the role of funders in the study. If the funders had no role, please include the statement: "The funders had no role in study design, data collection and analysis, decision to publish, or preparation of the manuscript."**

**Response:** We have included the statement: "The funders had no role in study design, data collection and analysis, decision to publish, or preparation of the manuscript."

**4. Confirm that all raw data required to replicate the study are included.**

**Response:** We confirm that all raw data required to replicate the study are included within the manuscript and the Supporting Information files.

**5. Figure 20 is referenced in the text but is missing. Please upload the figure or remove references to it.**

**Response:** We have removed all references to Figure 20, as it is not relevant to the revised manuscript.

**6. Please include your tables as part of the main manuscript and remove individual files. Supplementary tables should remain as "supporting information" files.**

**Response:** All tables have been incorporated into the main manuscript, and supplementary tables have been retained as separate supporting information files.

**Responses to Reviewer #1**

**1. The study lacks novelty and does not offer significant advancements beyond existing literature.**

**Response:** We appreciate this critical observation. While the use of nanoparticles to enhance antibiotic efficacy has been explored, our study provides several novel contributions:

**Quantification of conjugation efficiency:** We quantified the extent of ampicillin conjugation with AgNPs, demonstrating that approximately four molecules of ampicillin bind per nanoparticle.

**Mechanistic insights:** The study provides mechanistic explanations for enhanced antibacterial activity, including protection of the β-lactam ring from degradation and increased biofilm penetration.

**Comprehensive evaluation:** We evaluated biofilm inhibition (MBIC), eradication (MBEC), and time-kill kinetics, which are less frequently studied in similar works.

**Cytotoxicity analysis:** Our study confirms that AgNP-ampicillin conjugates exhibit >95% Vero cell viability, highlighting their safety.

These novel aspects have been emphasized in the **Introduction** and **Comparative Discussions Existing Literature** sections.

**Responses to Reviewer #2**

**1. The abstract is difficult to interpret due to numerical results. Simplify the presentation.**

**Response:** The abstract has been rewritten to provide a concise summary of key findings, avoiding excessive numerical data.

**2. Keywords are too specific (e.g., MIC, MBC, MBIC, MBEC).**

**Response**: Keywords have been revised to broader terms, such as "Antibiotic resistance," "Silver nanoparticles," and "Biofilm inhibition."

**3. Clarify whether AgNPs have antimicrobial properties on their own.**

**Response**: This has been clarified in the Background section. AgNPs exhibit inherent antimicrobial activity, which is enhanced when conjugated with ampicillin.

**4. The source of bacterial strains and biofilm formation methods are missing.**

**Response**: Details about bacterial strains, their susceptibility profiles, and biofilm formation methods have been included in the Materials and Methods section.

**5. Do you have stability, protein corona, or release assays?**

**Response**: While this study did not include long-term stability or protein corona assays due to resource constraints. However, we have added the zeta potential distribution curve of AgNP-ampicillin conjugates in **Figure 5B**.

**Responses to Reviewer #3**

**1. Microbiological methods are missing in the abstract.**

**Response**: The abstract has been updated to briefly mention the microbiological methods used.

**2. Tables and figures are missing.**

**Response**: All missing tables and figures have been included in the revised manuscript.

**3. Provide more details about bacterial strains, ethics statement, and susceptibility profiles.**

**Response**: Detailed information about bacterial strains and their susceptibility profiles has been added to the Materials and Methods section. An ethics statement has also been included.

**4. Define brands of culture media and clarify checkerboard assay concentrations.**

Response: Brands of culture media have been specified, and details of the checkerboard assay concentrations have been added.

**Responses to Reviewer #4**

**1. Revise the Introduction to focus on the clinical significance of MRSA.**

**Response**: The Introduction has been revised to emphasize the clinical importance of MRSA and its intrinsic resistance to ampicillin.

**2. Address concerns about ampicillin solubility during synthesis.**

**Response**: The methodology has been clarified to address potential issues with ampicillin solubility. Carboxyl-functionalized ampicillin with amine-functionalized AgNP using APTES has a better solubility profile in ethanol and remains in the supernatant, while the unreacted ampicillin was removed via centrifugation.

**3. Include statistical analyses for key experiments.**

**Response**: Statistical analyses, including one-way ANOVA and Tukey's post-hoc tests, have been performed and included in the revised manuscript.

**4. Provide a supplementary table for FIC values and clarify synergy classification.**

**Response**: A table with FIC values has been added, and the classification of synergy has been clarified in the Results section.

**Responses to Reviewer #5**

**General Notes**

**1. Mechanism**

**Comment**: The mechanism of action for the AgNP-ampicillin conjugate is not addressed.

**Response**: We have added a detailed discussion of the antimicrobial mechanism in the **Results and Discussion** section (Section 3.6). This includes the role of reactive oxygen species (ROS) generation, bacterial membrane disruption, enhanced biofilm penetration, and protection of the β-lactam ring from β-lactamase activity.

**2. Toxicity**

**Comment**: Potential cytotoxicity of the conjugates is not discussed.

**Response**: Cytotoxicity analysis was performed on Vero cells and included in the revised manuscript (Section 3.4). The results demonstrate >95% cell viability for AgNP-ampicillin conjugates, confirming minimal toxicity compared to standalone AgNPs.

**3. In vivo Testing**

**Comment**: The study lacks in vivo testing to validate translational potential.

**Response**: While this study is limited to in vitro assays, we have acknowledged this limitation in the **Conclusion** section and emphasized the need for future in vivo studies to evaluate the therapeutic potential of AgNP-ampicillin conjugates.

**4. Stability**

**Comment**: Long-term stability of the conjugates is not discussed.

**Response**: Preliminary stability data showing the zeta potential of -35.7 mV and particle size consistency over 30 days have been included (Section 3.1.4). We have also acknowledged the necessity for further studies on long-term stability under diverse storage conditions.

**Specific Comments**

**1. Title**

**Comment**: The title can be improved and made concise.

**Response**: The title has been revised to:

**"*Enhanced Antibacterial Activity of Ampicillin-Conjugated Silver Nanoparticles Against Methicillin-Resistant Clinical Isolates*"**

**2. Abstract**

**Comment**: The abstract is lengthy and includes numerical data.

**Response**: The abstract has been rewritten to be concise, avoiding excessive numerical data. A note on the need for further studies (e.g., in vivo efficacy) has also been added.

**3. Background**

**Comment**: Contains redundant information about multiple nanoparticles and lacks focus on AgNP-ampicillin.

**Response**: The **Background** section has been streamlined to focus on silver nanoparticles, ampicillin, and their synergy. Redundant information about other nanoparticles has been removed.

**4. Materials and Methods**

**Comment**: Several methodological details are missing (e.g., purity of chemicals, sonication time, storage conditions).

**Response**: The **Materials and Methods** section has been revised to include:

- Purity and grade of chemicals (e.g., "analytical grade," ≥99%).
- Sonication time and conditions (5 minutes, 40 kHz, 150 W).
- Details on nanoparticle storage (2–8°C in ethanol) after synthesis.

**5. Results and Discussion**

**Comment**: Mechanistic insights and interpretations are insufficient.

**Response**: The **Results and Discussion** section has been expanded to include:

- Mechanistic insights into the synergy between AgNPs and ampicillin (e.g., ROS generation, biofilm penetration).
- Comparisons with existing literature to highlight the novelty of this study.

**6. Figures and Tables**

**Comment**: Ensure all figures and tables are included and labeled.

**Response**: All figures and tables mentioned in the text are included in the revised manuscript with proper labels and legends.

**7. Conclusion**

**Comment**: The conclusion is vague and lacks specificity about key findings and limitations.

**Response**: The **Conclusion** section has been rewritten to summarize key findings (e.g., quantified conjugation efficiency, enhanced biofilm eradication, minimal cytotoxicity) and emphasize limitations (e.g., lack of in vivo testing).

I hope that my responses adequately address the reviewers' concerns and demonstrate my commitment to improving the manuscript. I believe that the revisions made have strengthened the paper, and I look forward to your favorable consideration for publication.

Thank you once again for your time and consideration.

Sincerely,

**Muhammad Salehuddin Ayubee**

Lecturer

Department of Pharmacy | Northern University Bangladesh

Dhaka-1230 | Bangladesh

Mobile: +88-01918659875

E-mail: msayubee@gmail.com
