# Supplementary material for: Synergistic antibacterial action of AgNP-ampicillin conjugates: Evading β-lactamase degradation in ampicillin-resistant clinical isolates
Source: PLoS One. 2025 Sep 9;20(9):e0331669. doi: 10.1371/journal.pone.0331669 (PMC12419620; doi:10.1371/journal.pone.0331669)
Supplement: Attachment — Submitted filename: Response_to_Reviewers_auresp_2.docx [file pone.0331669.s005.docx]

**Response to Reviewer #1**

**Comment 1:** Please clarify whether the MRSA strains used are clinical isolates and provide relevant source/ethics information.

**Response:** We appreciate the reviewer’s comment. The requested information regarding the MRSA strains is already included in **Table 2** of the manuscript, which specifies that these are clinical isolates. Additionally, an ethical statement is provided in the **Ethics Statement** section of the manuscript, confirming that the isolates were anonymized and collected during routine diagnostic microbiology procedures, with ethical approval obtained from the Ethical Review Committee, Faculty of Pharmacy, University of Dhaka **(Ref. No. Fa. Ph. E/128/A/22)**. To ensure clarity, we have cross-referenced this information in the relevant sections of the manuscript.

**Comment 2:** Statistical methods should be clearly stated in figure legends, and significance markers (e.g., p-values) should be consistently included.

**Response:** We have revised the manuscript to ensure that the statistical methods are explicitly mentioned. P-values have been added directly into tables where statistical tests were performed (e.g., Table 5 for disk diffusion results). Replicate data have been replaced with means and standard deviations, as per Reviewer #4’s suggestion, to improve clarity.

**Comment 3:**

The proposed mechanism via molecular docking is interesting but should be clearly framed as a hypothesis, not confirmed evidence.

**Response:** Thank you for highlighting this important point. We agree that the molecular docking results should be framed as a hypothesis rather than confirmed evidence. As such, we have revised the relevant sections of the "Results and Discussion" and "Conclusion" in the manuscript to clearly communicate that the docking results provide a **hypothetical mechanism** for the protection of ampicillin from β-lactamase degradation. Specifically, the molecular docking data are now discussed as a **computational prediction** that requires experimental validation through enzymatic assays or structural studies.

**Comment 4:**

Consider briefly discussing limitations such as the lack of in vivo validation or long-term stability testing.

**Response:** We acknowledge the reviewer’s suggestion and have incorporated a **Limitations** subsection in the **Discussions** section of the manuscript to address this concern. The limitations now clearly outline the gaps in the current study and suggest areas for future research.

**Response to Reviewer #2**

**Major Comment 1:** The approach is not novel at all; thus, the authors must clearly demonstrate why this work is different and discuss that in line with previous works.

**Response:** We have extensively revised the **Background** section to clearly articulate the novelty of our study and differentiate it from prior works. Specifically, we have highlighted the following unique contributions:

1. **Accurate Quantification of Conjugation Efficiency:** Prior studies have not quantified the conjugation efficiency of AgNP-antibiotic conjugates. This study reports a conjugation efficiency of 57.7%, with approximately four ampicillin molecules conjugated per AgNP.
2. **Mechanistic Insights:** We used molecular docking to propose a mechanism by which AgNP-ampicillin conjugates protect ampicillin from β-lactamase degradation, which has not been explored in previous research.
3. **Comprehensive Antibiofilm Assessment:** Unlike previous studies that focused on planktonic cells, this study evaluates antibiofilm activity through MBIC and MBEC assays.
4. **Mutant Prevention Studies:** We conducted mutant prevention concentration (MPC) assays to assess the conjugates’ ability to suppress resistant mutants, which is a critical but underexplored aspect of nanoparticle-antibiotic research.
5. **Cytotoxicity Evaluation:** We evaluated the cytotoxicity of the conjugates on Vero cells, demonstrating >95% cell viability, addressing safety concerns associated with standalone AgNPs.

These points are now explicitly stated in the revised **Background** section (Page 3-4).

**Major Comment 2**: Some assays require further description, and the sequence of presentation must be reconsidered.

**Response:** The revised manuscript offers a clearer and more systematic presentation of the experimental methods, addressing concerns about insufficient descriptions and sequence.

**Major Comment 3**: Checking All Abbreviations

**Response:** All abbreviations were defined at first usage in the text and listed under a dedicated "Abbreviations" section. For instance, "MBIC," "MBEC," and "FIC" were properly defined.

**Major Comment 4**: The "Results and Discussion" section is confusing, lacks a proper discussion, and mixes results and discussions. Consider separating results and discussion or re design the full section.

**Response:** The results and discussion section is separated to improve clarity and distinguish between results and interpretations.

The "Results" section now focuses solely on presenting experimental findings with clear subsections (e.g., chemical characterization, microbiological characterization, cytotoxicity).

A separate "Discussion" section contextualizes the results, compares findings with prior studies, and highlights advancements (e.g., β-lactamase protection, biofilm eradication, reduced cytotoxicity), and limitations.

**Minor Comments**

**0. Title**

**Reviewer Comment:** The title is too long and too descriptive.

**Response:** We thank the reviewer for pointing out the need for a more concise and precise title. In response, we have revised the title to better reflect the focus of the work while avoiding unnecessary length and excessive detail. We propose the following options for the title:

1. **Long Title:**
   *“Synergistic Antibacterial Action of AgNP-Ampicillin Conjugates: Evading β-Lactamase Degradation in Ampicillin-Resistant Clinical Isolates”*
   This title emphasizes the synergistic antibacterial effects, the β-lactamase protection mechanism, and the clinical relevance of the study.
2. **Short Title:**
   *“Multi-Mechanistic Antibacterial Evaluation of AgNP-Ampicillin Conjugates Against Ampicillin-Resistant Bacteria”*
   This shorter title highlights the evaluation of multiple antibacterial mechanisms while maintaining focus on the conjugates’ impact on resistant clinical isolates.

**1. Abstract
Reviewer Comment:**
a) “zone of inhibition” is not scientific at all. Change this expression.
b) “proper binding efficiency” or conjugation efficiency?
c) The use of statistics in the abstract to show the antibacterial potential of the nanoparticles makes it difficult to easily understand which molecule is the best.
**Response:**

a) “zone of inhibition” is replaced by “inhibition area” in the abstract

b) “proper binding efficiency” is replaced by “conjugation efficiency”

c) Statistical details have been simplified to focus on comparative performance, highlighting the enhanced efficacy of AgNP-ampicillin conjugates.

**2) Background**

**2.1 Reviewer Comment (Page 3):**

a) Add a brief sentence on why vancomycin resistance is terrible.

b) The formation of biofilms also makes some bacterial cells more dormant, preventing the use of certain antibiotics that require active bacteria to be effective. Add a sentence on this matter.

**Response:** Thank you for this suggestion. We have revised the Background section to include a sentence emphasizing the severity of vancomycin resistance. We have also included a statement on how biofilm formation promotes dormancy, preventing the use of certain antibiotics.

**2.2 Reviewer Comment (Page 4):**

a) New strategies to improve efficiency by increasing accumulation in the infectious site? What is the real advantage of using nanoparticles since the antibiotic is the same? Is the antimicrobial activity of nanoparticles itself?

b) Add some examples of the problems associated with AgNPs.

c) The authors state that recent studies “have investigated the conjugation of AgNPs with ampicillin against multidrug-resistant bacteria.” Thus, subsequently, in the Background section, the authors must explain the novelty of the current study.

**Response:**

a) We have clarified the advantages of using nanoparticles and their intrinsic antimicrobial properties in the revised Background section. The updated text now includes:

“Nanoparticles serve both as antimicrobial and a drug delivering system due to their small size. MNPs can penetrate bacterial cell walls and biofilms effectively, and the inherent bactericidal activity through generation of reactive oxygen species (ROS) works complementary to the antibacterial effect of antibiotics. These properties make nanoparticles a promising solution to address resistance mechanisms, particularly when conjugated with existing antibiotics to maximize synergistic effects.”

b) We have revised the Background section to include examples of challenges associated with AgNPs.

c) We have expanded the Background section to clearly articulate the research gap and novelty of the current study. The revised text highlights the unique contributions of our work:

"However, prior research has largely overlooked the cytotoxicity of these conjugates in mammalian cell lines, which is a key determinant for their safety and clinical applicability. Another critical gap in previous research is the lack of mutant prevention studies, which are essential for evaluating the potential of antimicrobial agents to suppress the emergence of resistant bacterial populations. Although the high antimicrobial potency of nanoparticle-antibiotic conjugates, involving antibiotics, is established, the accurate quantification of ampicillin conjugated per AgNP, which is critical for understanding the stability, have not been fully elucidated. Moreover, mechanistic insights into how these conjugates protect antibiotics from β-lactamase activity and inhibit biofilm, remain unexplored."

**2.3 Reviewer Comment (Page 5, a):** The problem with nanoparticle conjugation must be stated to demonstrate the importance of the current study. For instance, if the lack of proper characterization has been a problem in other papers, thus the “accurate quantification” is a plus in this work. In addition, if other works did not provide the mechanism of action, this “mechanistic insight” is a plus in this work. The authors must clearly state what is different and novel in this work; otherwise, it lacks novelty.

**Response:** We have revised the Background section to explicitly state the problems with nanoparticle conjugation in prior studies and how our work addresses these limitations. Articulated the novelty of the current study by addressing gaps in prior research (e.g., conjugation efficiency quantification, mechanistic insights, antibiofilm activity, cytotoxicity evaluation, and mutant prevention studies).

**3) Materials and Methods**

**3.1 Reviewer Comment (Page-8):**

a) Which method used for quantification is novel?

b) What is the relevance of mentioning SPR herein? Did the authors measure binding affinities or something similar? Why did you use SPR?

**Response:**

a) We have clarified in the revised manuscript that the novelty lies in the integration of UV-Vis spectroscopy (To quantify the amount of silver present in the conjugate by the conjugation efficiency equation) with thermogravimetric analysis (TGA) (comparing the degradation pattern of pure ampicillin and conjugate), and EDX (To quantify the amount of silver present in the conjugate) to quantify conjugation efficiency. This approach ensures accuracy and reproducibility, offering a significant improvement over previous reports, where conjugation efficiency was often not quantified or was indirectly inferred. The corresponding text has been included in Section 2.2.5 (Quantification of unreacted ampicillin in the supernatant).

b) The mention of surface plasmon resonance (SPR) is relevant because it confirms the successful synthesis of silver nanoparticles (AgNPs) by identifying their characteristic absorption peak in the UV-Vis spectrum (380–440 nm). While we did not measure binding affinities directly using SPR, its inclusion validates the structural integrity and stability of the nanoparticles, which is essential for their subsequent functionalization and conjugation with ampicillin. This reasoning will be clarified in Section 2.3.1 (UV-Vis Spectroscopy).

**3.2 Reviewer Comment (Page 11):** a) Where are these strains coming from? Distributor? Hospital? Biobank? The authors have some information on Table 2. Do you have access to the clinical data of these patients? It would be very interesting to see the primary disease, response to treatment, and other things.

**Response:**

The bacterial strains used in this study were sourced from the microbiology laboratory of Mohakhali TB Hospital and BIRDEM Medical College. These strains were anonymized and collected as part of routine diagnostic microbiology procedures. Ethical approval for their use was obtained (Ref. No. Fa. Ph. E/128/A/22).

We do not have access to detailed clinical data of the patients, such as primary disease, response to treatment, or other demographic information, as it falls outside the scope of this study and ethical limitations. However, we agree that such data could provide valuable insights and will consider this in future studies. This clarification will be added under Section 2.4.1 (Collection of Clinical Isolates).

**3.3 Reviewer Comment (Page 12):**

a) What is the relevance of bacteria characteristics in the materials and methods? Move this very relevant information to the discussion.

b) Where is the information on the biofilm formation?

**Response:**

a) We appreciate the reviewer’s suggestion and agree that the bacterial characteristics are more relevant to the Discussion section, under a new subheading titled "Relevance of Selected Bacterial Strains".

b) The information on biofilm formation is present under Section 2.4.5 (MBIC and MBEC assay), which includes details on how biofilms were formed and evaluated.

**3.3 Reviewer Comment (Page 14):**

a) Where is the information on the culturing conditions of Vero and its source?

b) Where is the description of the cytotoxicity assay?

**Response:**

a) The culturing conditions of Vero cells and their source is included in Subsection 2.5.1 and Subsection 2.5.2.

b) Cytotoxicity assay procedure were briefly described in Subsection 2.5.3 (Cytotoxicity assay of test samples) (Page 21). Detail experimental procedures were included in the supplementary information (Cytotoxicity Assay Procedure).

**3.4 Reviewer Comment (Page 15):** a) Statistical analysis comes usually at the end of the materials and methods section.

**Response:** Since different statistical parameter used for different assay, they are mentioned in the respective assay for relevance and clarity. For example, One-way ANOVA and Tukey's Post Hoc test procedure were outlined in the disk diffusion assay (Page 16).

**3.5 Reviewer Comment (Page 21):** a) Here, the authors have the cytotoxicity assay but they have mentioned it before. Please re-arrange the materials and methods section to be easier to interpret.

**Response:** Cytotoxicity Assay was divided into three subsections to ensure clarity. The title of subheading 2.4.2 (General preparation of media and controls for microbiological assays) was modified to avoid confusion with the Vero cell culture and Cytotoxicity Assay. Additionally, the cytotoxicity assay method was outlined before the microbiological assay.

**4) Results**

**4.1 Reviewer Comment (Page 22):** a) The reviewer does not understand why SPR is here, the assay is not mentioned in the materials and methods, concerning the chip, the solvents, the concentrations.

**Response:** The rationale of SPR will be clarified in the materials and methods- Section 2.3.1 (UV-Vis Spectroscopy)

**4.2 Reviewer Comment:**

**(Page 23):**

a) These NPs are very heterogeneous! Average size of 67? The results show at least three relevant peaks… What is the PDI? That value must be presented.

b) Why is the size increasing so much? 500 nm is huge! Do you know the in vivo consequences of that size?

**(Page 24):** a) Why is the size increasing so much?

**Response:**

**(Page 23a):** AgNPs was averaged 67.34 nm in diameter with polydispersity index (PDI) of 0.316 indicating moderate polydispersity (Section 3.1.1). In order to increase the homogeneity, the synthesized AgNPs were silica coated and amine functionalized, which resulted in uniform amine functionalized AgNPs providing single peak with PDI of 0.092 (Section 3.1.2). The information on PDI value will be included in the manuscript (Section 2.3.2). Detailed information will be made available in the supporting information.

**(Page 23b and 24a):** The size of particles increased after amine functionalization and further size enhancement was observed after the conjugation. The conjugated product could act as a prodrug by hydrolyzing into four ampicillin molecules at the infection site and improving the local concentration of the antibiotics, particularly for wound or localized infections. Subsequently, the functionalized AgNP would be taken up by liver cells, transported into bile ducts, enter the intestines, and then eliminated from the body through feces (1, 2).

Studies show that antibiotic-loaded nanoparticles resulting in larger particle size, provide more sustained and targeted drug release, resulting in enhanced bacterial killing and improved wound healing compared to free antibiotics (3). In animal models, such conjugates reduced bacterial counts at infection sites more effectively than free drugs and promoted faster tissue recovery (4). Besides, due to their rapid clearance and localized accumulation, antibiotic conjugated nanoparticles with larger particle size tend to have lower systemic toxicity compared to the smaller one (5).

***References:***

1.	Longmire M, Choyke PL, Kobayashi H. Clearance properties of nano-sized particles and molecules as imaging agents: considerations and caveats. Nanomedicine (Lond). 2008;3(5):703-17.

2.	Poon W, Zhang YN, Ouyang B, Kingston BR, Wu JLY, Wilhelm S, et al. Elimination Pathways of Nanoparticles. ACS Nano. 2019;13(5):5785-98.

3.	Hussain S, Joo J, Kang J, Kim B, Braun GB, She ZG, et al. Antibiotic-loaded nanoparticles targeted to the site of infection enhance antibacterial efficacy. Nat Biomed Eng. 2018;2(2):95-103.

4.	Taheri M, Arabestani MR, Kalhori F, Soleimani Asl S, Asgari M, Hosseini SM. Antibiotics-encapsulated nanoparticles as an antimicrobial agent in the treatment of wound infection. Front Immunol. 2024;15:1435151.

5.	Alexis F, Pridgen E, Molnar LK, Farokhzad OC. Factors affecting the clearance and biodistribution of polymeric nanoparticles. Mol Pharm. 2008;5(4):505-15.

**4.3** **Reviewer Comment (Page 26):** a) It is odd to have the mechanism of synthesis stated like this in the manuscript…

Response: A new paragraph is added to explain the synthesis reactions and the title of the Section 3.2 is changed to “AgNP-ampicillin Synthesis Reaction” for clarity.

**4.4 Reviewer Comment (Page 34)**: a) Cytotoxicity towards Vero must come first.

**Response:** The cytotoxicity assay results will be repositioned before the microbiological assay in the manuscript. We believe this adjustment enhances the logical flow of the manuscript and addresses the reviewer’s concern effectively.

**4.5 Reviewer Comment (Page 34)**: a) This mechanism part is relevant but, in the reviewer’s, opinion is in the wrong place. It must be discussed throughout the text and not like this.

**Response**: We thank the reviewer for this valuable comment. In the revised manuscript, we have integrated the discussion of the antimicrobial mechanism of AgNP-ampicillin throughout the text instead of presenting it in different subsections.

**Response to Reviewer #4**

**Major Comments**

1. **Comment:** In the Materials and Methods section, the use of the adjective “methicillin-resistant” to describe bacteria other than staphylococci is highly unusual. Please replace this term with “ampicillin-resistant,” which would be more accurate.

**Response:** The term "methicillin-resistant" has been replaced with "ampicillin-resistant" throughout the manuscript when referring to bacterial isolates other than staphylococci. This aligns with the reviewers' suggestion for accuracy.

2. **Comment:** Regarding the interpretation of FIC values, please use only the following reference: https://academic.oup.com/jac/article-abstract/52/1/1/930000. This is the only source that originally defines these values correctly. Remove all other references and reinterpret your results accordingly.

**Response:** The manuscript now references the recommended source (<https://academic.oup.com/jac/article-abstract/52/1/1/930000?redirectedFrom=fulltext>) for interpreting FIC values. The FIC data have been reanalyzed and interpreted accordingly.

3. **Comment:** The statistical methods are described in excessive detail, which often becomes unnecessarily cumbersome. Moreover, the results are repetitive, as they are presented both in the text and in the tables.

**Response:** Statistical details in the revised manuscript have been streamlined. Unnecessary complexity has been reduced, and tables now summarize key findings instead of presenting repetitive statistics.

4. **Comment:** Many of these results could be better represented graphically, yet the authors have chosen to use multiple Tables (e.g., Table 5) or Tables plus graphs (e.g., Table 8 and Figure 9).

**Response:** In the revised manuscript, several results previously presented as tables and text have been reorganized. Graphical representations have been used where appropriate to avoid redundancy. However, the previous review recommended table presentation for detailed insight. So, less important tables and figures are included in the supplementary information.

5. **Comment:** Several figures could be grouped together to reduce the overall number of figures in the manuscript.

**Response:** Some figures in the revised manuscript have been grouped (e.g., Figures 11 and 12) in the revised manuscript, reducing the total number of figures.

6. **Comment:** In Table 5, please remove the replicate data, and present only the mean and standard deviation for each bacterial species. Also, include the p-value within the table and remove the contrast statistics. Do something similar with the remaining tables.

**Response:** The revised manuscript removes replicate data from tables and instead includes averages (mean, standard deviation) for each bacterial species. P-values are now included in the table, and contrast statistics have been removed.

7. **Comment:** Regarding the cytotoxicity assays, how many biological replicates were performed? Why are these results presented in a table instead of as a graph?

**Response:** Three biological replicates were performed in the assay. Cytotoxicity results have been presented in table for clarity.

**Minor Comments**

**1. Comment:** The “spp.” in Enterobacter spp. and Serratia spp. should not be italicized.

**Response:** The term "Enterobacter spp." and "Serratia spp." is no longer italicized.

**2. Comment:** Enterobacter aerogenes has been reclassified as Klebsiella aerogenes. Please update this in the manuscript.

**Response:** "Enterobacter aerogenes" has been updated to "Klebsiella aerogenes" throughout the manuscript, reflecting its current taxonomic classification.
