# Supplementary material for: Synergistic antibacterial action of AgNP-ampicillin conjugates: Evading β-lactamase degradation in ampicillin-resistant clinical isolates
Source: PLoS One. 2025 Sep 9;20(9):e0331669. doi: 10.1371/journal.pone.0331669 (PMC12419620; doi:10.1371/journal.pone.0331669)
Supplement: Attachment — Submitted filename: Response to Reviewer.docx [file pone.0331669.s006.docx]

**Response to Reviewer #2:**

We sincerely appreciate your kind feedback and are grateful for your positive evaluation of the revised manuscript. We are pleased to know that we have adequately addressed your comments. Thank you for your support.

**Response to Reviewer #4:**

We thank you for your thoughtful and constructive feedback, which has contributed significantly to improving the quality of our manuscript. We have carefully addressed all your concerns and made the necessary revisions.

**1.** **Comment:** Definition of the MBEC assay protocol

We appreciate your clarification regarding the MBEC assay and agree that the original definition did not accurately reflect the concept of biofilm eradication. In response to your comment, we have revised the manuscript to provide the correct definition. Specifically, we have updated the text to read:

The MBEC was determined to be the lowest concentration of the test sample that can kill all microorganisms within a biofilm, which is associated with biofilm disaggregation effect.

**2. Comment:** Clarification on Tables 6 and 7 – Meaning of “0”

We appreciate your observation regarding the “0” value in Tables 6 and 7, which actually represents that we didn’t find any growth inhibition with the tested concentrations of sample.

We acknowledge that this could be misleading and are grateful for your suggestion to clarify the data. In response, we have revised the tables and replaced the “0” value with “>100 [highest tested concentration]” to more accurately reflect that no tested concentration was effective and more concentrated sample is required for growth inhibition.
